# Acute antiarrhythmic effects of SGLT2 inhibitors–dapagliflozin lowers the excitability of atrial cardiomyocytes

**DOI:** 10.1007/s00395-023-01022-0

**Published:** 2024-01-03

**Authors:** Amelie Paasche, Felix Wiedmann, Manuel Kraft, Fitzwilliam Seibertz, Valerie Herlt, Pablo L. Blochberger, Natasa Jávorszky, Moritz Beck, Leo Weirauch, Timon Seeger, Antje Blank, Walter E. Haefeli, Rawa Arif, Anna L. Meyer, Gregor Warnecke, Matthias Karck, Niels Voigt, Norbert Frey, Constanze Schmidt

**Affiliations:** 1 Department of Cardiology, Medical University Hospital Heidelberg, Im Neuenheimer Feld 410, 69120 Heidelberg, Germany; 2 DZHK (German Center for Cardiovascular Research), Partner site Heidelberg/Mannheim, University of Heidelberg, Im Neuenheimer Feld 669, 69120 Heidelberg, Germany; 3 HCR, Heidelberg Center for Heart Rhythm Disorders, University Hospital Heidelberg, Im Neuenheimer Feld 410, 69120 Heidelberg, Germany; 4 Institute of Pharmacology and Toxicology, University Medical Center Göttingen, Robert Koch Strasse 42a, 37075 Göttingen, Germany; 5 DZHK (German Center for Cardiovascular Research) Partner Site Göttingen, Robert Koch Strasse 42a, 37075 Göttingen, Germany; 6 Cluster of Excellence “Multiscale Bioimaging: from Molecular Machines to Networks of Excitable Cells” (MBExC), University of Göttingen, Robert Koch Strasse 40, 37075 Göttingen, Germany; 7 Department of Clinical Pharmacology and Pharmacoepidemiology, University Hospital Heidelberg, Im Neuenheimer Feld 410, 69120 Heidelberg, Germany; 8 Department of Cardiac Surgery, University Hospital Heidelberg, Im Neuenheimer Feld 410, 69120 Heidelberg, Germany

**Keywords:** SGLT2 inhibitors, Dapagliflozin, Atrial action potential, Na_V_1.5, Atrial fibrillation

## Abstract

In recent years, SGLT2 inhibitors have become an integral part of heart failure therapy, and several mechanisms contributing to cardiorenal protection have been identified. In this study, we place special emphasis on the atria and investigate acute electrophysiological effects of dapagliflozin to assess the antiarrhythmic potential of SGLT2 inhibitors. Direct electrophysiological effects of dapagliflozin were investigated in patch clamp experiments on isolated atrial cardiomyocytes. Acute treatment with elevated-dose dapagliflozin caused a significant reduction of the action potential inducibility, the amplitude and maximum upstroke velocity. The inhibitory effects were reproduced in human induced pluripotent stem cell-derived cardiomyocytes, and were more pronounced in atrial compared to ventricular cells. Hypothesizing that dapagliflozin directly affects the depolarization phase of atrial action potentials, we examined fast inward sodium currents in human atrial cardiomyocytes and found a significant decrease of peak sodium current densities by dapagliflozin, accompanied by a moderate inhibition of the transient outward potassium current. Translating these findings into a porcine large animal model, acute elevated-dose dapagliflozin treatment caused an atrial-dominant reduction of myocardial conduction velocity in vivo. This could be utilized for both, acute cardioversion of paroxysmal atrial fibrillation episodes and rhythm control of persistent atrial fibrillation. In this study, we show that dapagliflozin alters the excitability of atrial cardiomyocytes by direct inhibition of peak sodium currents. In vivo*,* dapagliflozin exerts antiarrhythmic effects, revealing a potential new additional role of SGLT2 inhibitors in the treatment of atrial arrhythmias.

**Supplementary Information:**

The online version contains supplementary material available at 10.1007/s00395-023-01022-0.

## Introduction

Sodium-glucose linked transporter 2 (SGLT2) inhibitors (SGLT2i), initially introduced as anti-hyperglycemic agents, gained major importance in the treatment of cardiological patients as they surprised with extensive clinical benefits beyond glycemic control. By now, dapagliflozin and empagliflozin are recommended and already commonly integrated in the standard pharmacotherapy of heart failure (HF) patients [25].

SGLT2i promote urinary glucose excretion by decreasing glucose reabsorption in the early proximal renal tubule [17]. Several large randomized trials investigating the impact of SGLT2i on patients with type 2 diabetes mellitus (T2DM) have consistently shown a decrease of HF-related hospitalizations and/ or a reduced risk of all-cause or cardiovascular mortality [26, 27, 33, 55, 58]. The DAPA-HF, EMPEROR-Reduced and EMPEROR-Preserved studies further revealed that dapagliflozin and empagliflozin exert beneficial effects on cardiovascular outcomes regardless of the presence or absence of diabetes [26, 31].

As SGLT2 is not relevantly expressed within the heart, different modes of action contributing to the cardioprotective effects are frequently discussed and focus mainly on cardiac metabolism, calcium handling and inflammatory processes. SGLT2i are thought to reduce adipose tissue and body weight and further increase circulating ketone levels, leading to improved mitochondrial function [11, 50]. Furthermore, SGLT2i prevent intracellular accumulation of sodium and resulting reductions in mitochondrial calcium levels due to several mechanisms, including attenuation of oxidative stress, decreased activity of the sarcolemma sodium-hydrogen exchanger 1 and inhibition of late inward sodium currents [15, 34, 48, 49]. Additionally, attenuation of inflammatory responses, which promote cardiac fibrosis, by SGLT2i has been observed [6]. On the molecular level, the regulation of cellular nutrient housekeeping has been identified as an important group of mechanisms underlying the protective effects on the cardiorenal system [30]. As of today, there is a consensus that the mechanisms are pleiotropic and collectively contribute to the beneficial cardiovascular effects.

Further studies and meta-analyses also indicated an association between SGLT2i treatment and a significantly reduced risk of atrial arrhythmias and sudden cardiac death [8, 14, 57]. There are complex interactions between atrial fibrillation (AF) and HF, as HF is a predisposing factor for AF while AF is known to be associated with worsening of outcomes in HF patients [24, 32, 56]. In general, AF is the most common sustained cardiac arrhythmia and relevantly contributes to population morbidity and mortality. However, there is still an urgent need for adequate pharmacotherapeutic treatment strategies. Therefore, it is of great interest to understand whether antiarrhythmic properties of SGLT2i might also contribute to their cardioprotective effects, in the context of, as well as independent of HF. Assessing the direct antiarrhythmic potential of SGLT2i might identify new additional indications and concepts for SGLT2i treatment.

To particularly assess the antiarrhythmic potential of SGLT2i, independent of the previously identified pleiotropic effects, it is crucial to understand direct molecular effects on cellular electrophysiological properties of the heart.

Here, we aimed to advance our understanding of direct effects of SGLT2i on atrial electrophysiology. We found that acute application of increased single-dose dapagliflozin suppresses action potential (AP) formation by directly inhibiting peak sodium currents in human atrial cardiomyocytes (CMs) in vitro, leading to both, cardioversion of acute AF episodes to sinus rhythm (SR) and rhythm control of persistent AF in a large-animal model in vivo. This indicates acute class I antiarrhythmic effects of dapagliflozin and forms the molecular basis for a potential new additional role of SGLT2i as antiarrhythmic agents. The combination of the previously identified pleiotropic chronic protective effects on the ventricles and the acute antiarrhythmic effects on atrial cardiomyocytes we newly discovered is remarkable, and might provide new options in combining chronic and acute SGLT2i treatment.

## Materials and methods

A detailed description of specific methods is provided in the Supplementary Materials and Methods section.

### Study design and ethics statement

A total of 36 patients (6 female; 30 male) with either SR (n = 21), paroxysmal or persistent AF (pAF; n = 9) or permanent /chronic AF (cAF; n = 6) undergoing open heart surgery for coronary artery bypass grafting, heart valve replacement /repair or maze procedures were included in the study. Written informed consent was given by all patients. The study protocol involving human tissue samples was approved by the responsible Ethics Committee of the Medical Faculty of Heidelberg University (Germany; S-017/2013) and was conducted in accordance with the 1964 Declaration of Helsinki. Human induced pluripotent stem cell (hiPSC) line UMGi014-C clone 14 (isWT1.14) was derived from dermal fibroblasts of a healthy male donor and experimental protocols were approved by the ethics committee of the University Medical Center Göttingen (10/9/15). All animal experiments were conducted in accordance with the Guide for the Care and Use of Laboratory Animals adopted by the US National Institutes of Health (NIH publication No. 86–23, revised 1985), with EU Directive 2010/63/EU, with the current version of the German Law on the Protection of Animals, and the ARRIVE guidelines. The study protocol was approved by the local Animal Welfare Committee (Regierungspräsidium Karlsruhe, Germany, reference numbers G-165/19; G-67/20; G-229/21). Acute AF was induced via right-atrial burst stimulation (400–1200 min^−1^) during EP studies. Induction of persistent AF in pigs was carried out by atrial burst stimulation via an implanted cardiac pacemaker (St. Jude Medical, St. Paul, MN, USA). To prevent tachycardia-induced heart failure, atrioventricular (AV) node ablation was performed under fluoroscopic guidance.

### Cellular electrophysiology

Human and porcine atrial myocytes were freshly isolated. Electrophysiological recordings were carried out using the whole-cell patch clamp configuration. Chinese hamster ovary (CHO) cells were transiently transfected with plasmid DNA encoding for Na_V_1.5 wild-type (WT) or the respective pore mutants. HiPSCs were differentiated in atrial- and ventricular-like hiPSC-derived cardiomyocytes (hiPSC-CMs) following previously published protocols [43]. Automated patch clamp (APC) experiments were performed using the SyncroPatch 384 APC System (Nanion Technologies, Munich, Germany) as previously described [44]. Electrical activity of hiPSC-CM monolayers was assessed using the Maestro Pro multi-electrode array (MEA) system (Axion Biosystems, Atlanta, USA) as described in the Supplemental Material online.

## Results

### Dapagliflozin alters the formation of APs in porcine atrial CMs

**Fig. 1:**
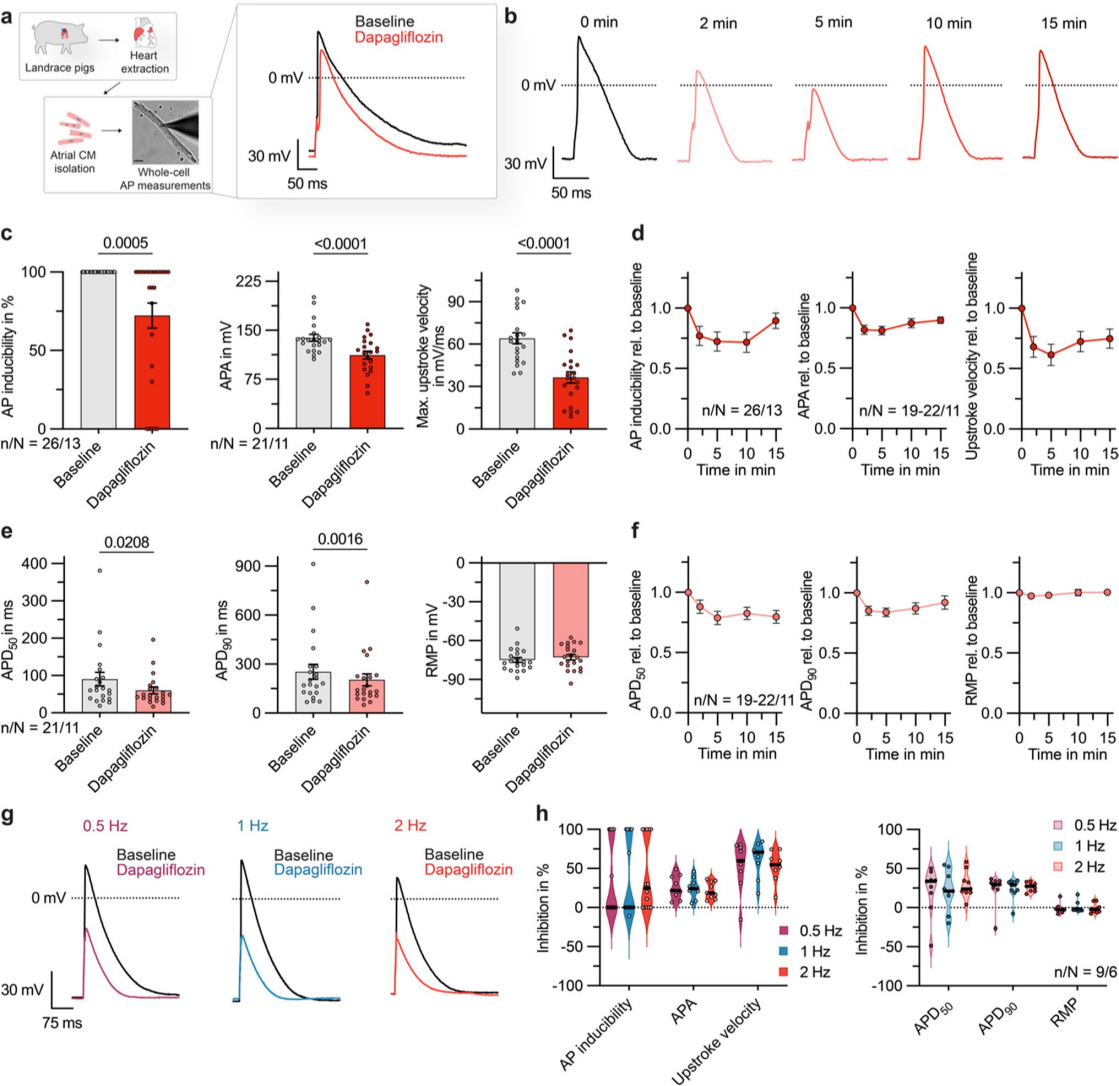
Effect of acute dapagliflozin treatment on action potential (AP) formation in isolated porcine atrial cardiomyocytes (CMs). **a** *Left:* Experimental protocol: fresh atrial tissue samples from N = 13 pigs were enzymatically digested. APs from n = 26 isolated CMs were recorded at baseline and after administration of dapagliflozin (100 µmol/L), using current clamp measurements in a whole-cell configuration with ruptured patches. The scalebar in the lower panel indicates 25 µm. Right: Representative AP recordings, obtained from a porcine atrial CM at baseline and after administration of dapagliflozin (100 µmol/L). **b** Representative AP recordings, under baseline conditions and in the time course after administration of dapagliflozin (100 µmol/L). **c** AP inducibility (percentage of pulses evoking an AP, out of 10 current pulses elicited at a rate of 0.5 Hz; n/N = 26/13), AP amplitude (APA) and maximum upstroke velocity of elicited APs (n/N = 21/11) at baseline and 5 min after application of dapagliflozin (100 µmol/L). **d** AP inducibility (n/N = 26/13), APA and maximum upstroke velocity (n/N = 19–22/11) relative to baseline values in the time course after administration of dapagliflozin (100 µmol/L; stimulation frequency = 0.5 Hz). **e** AP duration at 50% and 90% repolarization (APD_50_, APD_90_) and resting membrane potential (RMP) at baseline and 5 min after application of dapagliflozin (100 µmol/L; n/N = 21/11; stimulation frequency = 0.5 Hz). **f** APD_50_, APD_90_ and RMP relative to baseline values in the time course after administration of dapagliflozin (100 µmol/L; n/N = 19–22/11; stimulation frequency = 0.5 Hz). **g** Representative APs recorded at stimulation frequencies of 0.5, 1, and 2 Hz are shown under baseline conditions and after administration of dapagliflozin (100 µmol/L). **h** Violin plots showing dapagliflozin effects on AP inducibility (n/N = 14/8) and AP parameters (n/N = 9/6) at 0.5, 1 and 2 Hz stimulation frequency. Unless otherwise stated, data are shown as mean ± SEM. P-values were derived from paired Student’s t-tests

To investigate whether dapagliflozin exerts direct effects on atrial CM electrophysiology, we recorded APs from isolated porcine atrial CMs under control conditions and after direct administration of dapagliflozin at a concentration of 100 µmol/L (Fig. 1a). Acute application of increased-dose dapagliflozin served to differentiate direct antiarrhythmic effects of dapagliflozin from the pleiotropic cardioprotective effects of chronic SGLT2i treatment. We found strong alterations in the formation and shape of atrial APs under dapagliflozin treatment. APs were elicited by 10 current pulses at a rate of 0.5 Hz and the percentage of pulses evoking an AP was significantly reduced after administration of dapagliflozin (Fig. 1b, c). At the same time, the AP amplitude (APA) and the maximum upstroke velocity of elicited APs were significantly reduced after administration of the SGLT2i (20.7 ± 3.6% and 38.7 ± 8.8%; Fig. 1c, d). AP durations at 50% or 90% repolarization (APD_50_ and APD_90_) were significantly shortened (21.3 ± 5.6% and 16.1 ± 3.6%), while resting membrane potentials (RMPs) did not show significant changes (Fig. 1e). A time-resolved evaluation of dapagliflozin effects on AP parameters showed a tendency towards attenuation of the effects 15 min after drug admission (Fig. 1b, d, f). Dapagliflozin effects on AP parameters were conserved among different stimulation frequencies (0.5, 1, and 2 Hz) (Fig. 1g, h and Fig. S1). In addition, there was no significant difference between CMs isolated from left or right atrial samples with respect to their electrical response to dapagliflozin treatment (Fig. S2). Dapagliflozin effects were conserved in CMs obtained from pigs with both SR and persistent AF (Fig. S3). However, especially the reduction in AP inducibility was significantly pronounced in AF pigs (Fig. S3). We conclude that dapagliflozin acutely results in a lowered excitability of atrial porcine CMs as well as a reduction of the amplitude and upstroke velocity of atrial APs.

### Dapagliflozin suppresses atrial AP formation in human native and hiPSC-derived CMs

**Fig. 2:**
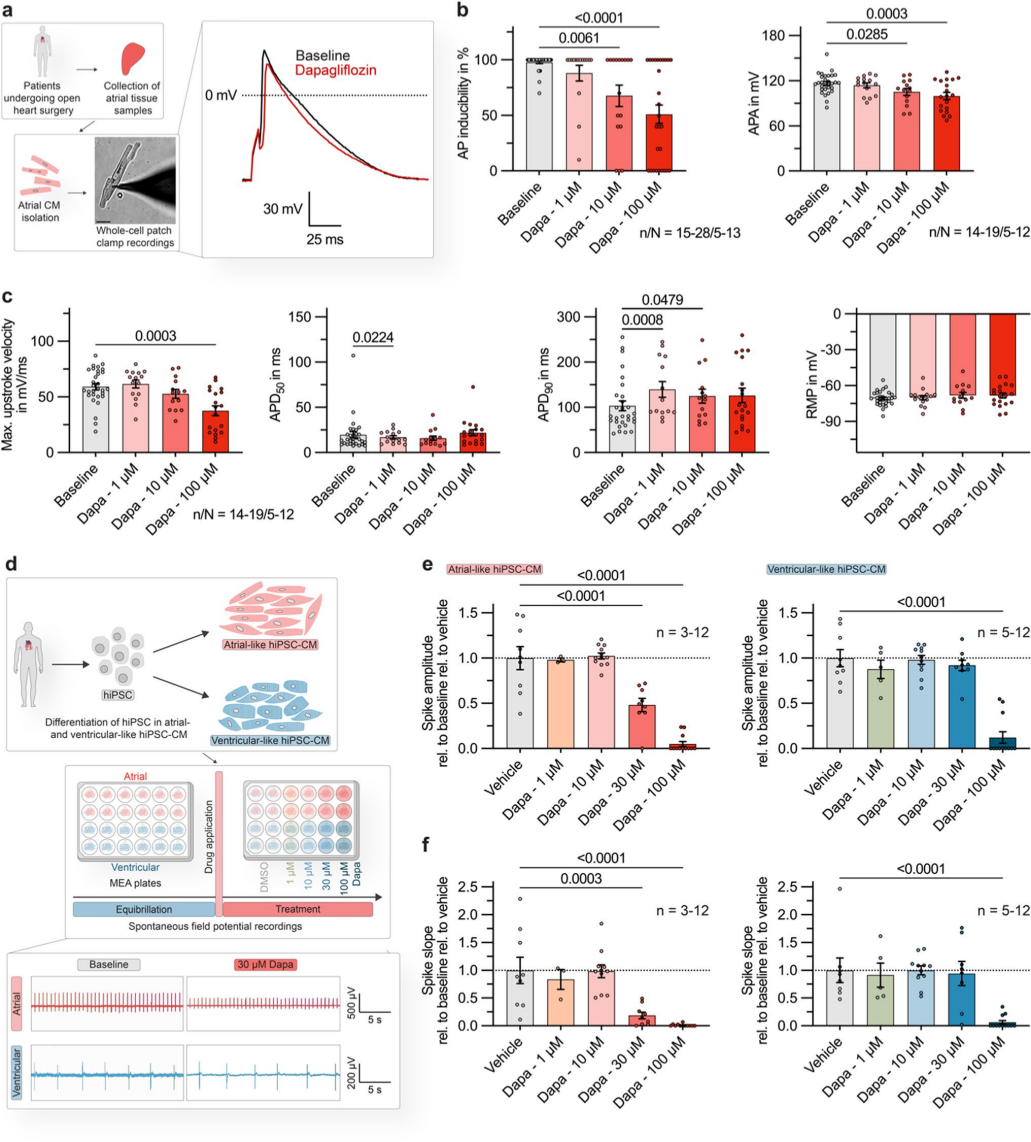
Direct electrophysiological effects of dapagliflozin on human atrial cardiomyocytes (CMs). **a** *Left:* Experimental protocol: fresh atrial tissue samples were obtained from N = 14 patients undergoing open heart surgery. APs from n = 34 isolated CMs were recorded at baseline and after administration of dapagliflozin at various concentrations (1, 10, 100 µmol/L), using current clamp measurements in a whole-cell configuration with ruptured patches. The scalebar in the lower panel indicates 25 µm. *Right:* Representative AP recordings, obtained from a human atrial CM at baseline and after administration of dapagliflozin (100 µmol/L). **b** AP inducibility (percentage of pulses evoking an AP, out of 10 current pulses elicited at a rate of 0.5 Hz; n/N = 15–28/5–13) and AP amplitude (APA) of elicited APs (n/N = 14–19/5–12) at baseline and 5 min after application of dapagliflozin (1, 10, 100 µmol/L). **c** Maximum upstroke velocity, AP duration at 50% and 90% repolarization (APD_50_, APD_90_) and resting membrane potential (RMP) in human atrial CMs under baseline conditions and 5 min after application of dapagliflozin (1, 10, 100 µmol/L; n/N = 14–19/5–12; stimulation frequency = 0.5 Hz). **d** Upper: Experimental protocol: human induced pluripotent stem cells (hiPSC) were differentiated in atrial- or ventricular-like hiPSC-derived CM (hiPSC-CM) and seeded on multi-electrode array (MEA) plates. Spontaneous field potentials were recorded under control conditions and after application of dapagliflozin at various concentrations (1, 10, 30, 100 µmol/L) or the vehicle. *Lower:* Representative field potential recordings, for atrial-and ventricular-like hiPSC-CM monolayers at baseline and 15 min after administration of dapagliflozin (30 µmol/L). **e** Relative spike amplitudes of field potentials recorded from atrial-(left) and ventricular-like (*right*) hiPSC-CM 15 min after application of dapagliflozin (1, 10, 30, 100 µmol/L; atrial: n = 3–12; ventricular: n = 5–12). **f** Relative spike slopes of field potentials recorded from atrial- (*left*) and ventricular-like (*right*) hiPSC-CM 15 min after application of dapagliflozin (1, 10, 30, 100 µmol/L; atrial: n = 3–12; ventricular: n = 5–12). If not indicated otherwise, data are shown as mean ± SEM. P-values were derived from ordinary one-way analysis of variance (ANOVA)

Next, we investigated whether dapagliflozin also affects the AP formation in human atrial CMs. Therefore, CMs were isolated from fresh atrial tissue samples obtained from patients undergoing open heart surgery (Table 1, Fig. 2a). In human atrial CMs, dapagliflozin caused a concentration-dependent reduction in AP inducibility, APA, and maximum upstroke velocity. AP inducibility and APA were significantly decreased after administration of dapagliflozin at 10 µmol/L (31.4 ± 9.8% and 9.1 ± 3.6%) and 100 µmol/L (48.7 ± 8.3% and 14.3 ± 3.1%; Fig. 2b). The maximum upstroke velocity was reduced from 59.2 ± 2.9 mV/ms at baseline to 52.8 ± 3.9 mV/ms under 10 µmol/L and 37.6 ± 4.5 mV/ms under 100 µmol/L dapagliflozin (Fig. 2c). The extent of reduction in AP inducibility, APA and upstroke velocity by dapagliflozin was consistent in human and porcine atrial CMs, demonstrating the reproducibility of the effect among different species. At the same time, the APD_90_ of human atrial CMs showed a mild prolongation upon dapagliflozin treatment while the RMP was not significantly altered (Fig. 2c). Of note, upon application of the solvent dimethyl sulfoxide (DMSO) no significant changes of AP parameters could be observed (Fig. S4).

**Table 1:** Baseline characteristics of study patients

	SR (n = 21)	pAF (n = 9)	cAF (n = 6)
Demographics			
Age, y (mean ± SEM)	63.40 ± 1.8	71.7 ± 2.1*	69.2 ± 3.0
Female, n (%)	1 (4.8)	3 (33.3)	2 (33.3)
Height, cm (mean ± SEM)	177.1 ± 1.5	171.2 ± 3.4	174.5 ± 4.0
Body weight, kg (mean ± SEM)	84.1 ± 2.8	77.6 ± 6.8	83.0 ± 7.6
BMI, kg/m^2^ (mean ± SEM)	26.8 ± 0.8	26.2 ± 1.7	27.2 ± 2.2
Echocardiography			
LVEF, % (mean ± SEM)	52.5 ± 2.8	43.7 ± 4.7	47.2 ± 5.4
LA diameter, mm (mean ± SEM)	40.9 ± 1.0	47.5 ± 3.0	49.2 ± 3.0
Valvular heart disease			
Aortic stenosis			
I	1 (4.8)	1 (11.1)	1 (16.7)
I–II	0 (0)	0 (0)	0 (0)
II	0 (0)	0 (0)	0 (0)
II–III	0 (0)	0 (0)	1 (16.7)
III	4 (19)	4 (44.4)	2 (33.3)
Aortic regurgitation			
I	2 (9.5)	3 (33.3)	1 (16.7)
I–II	1 (4.8)	0 (0)	0 (0)
II	1 (4.8)	0 (0)	0 (0)
II–III	0 (0)	0 (0)	0 (0)
III	1 (4.8)	0 (0)	0 (0)
Mitral stenosis			
I	0 (0)	1 (11.1)	0 (0)
I–II	0 (0)	0 (0)	0 (0)
II	0 (0)	0 (0)	0 (0)
II–III	0 (0)	0 (0)	0 (0)
III	0 (0)	0 (0)	0 (0)
Mitral regurgitation			
I	13 (61.9)	5 (55.6)	2 (33.3)
I–II	2 (9.5)	0 (0)	1 (16.7)
II	0 (0)	0 (0)	0 (0)
II–III	0 (0)	0 (0)	0 (0)
III	1 (4.8)	1 (11.1)	1 (16.7)
Tricuspid valve regurgitation			
I	5 (23.8)	2 (22.2)	3 (50)
I–II	1 (4.8)	2 (22.2)	1 (16.7)
II	1 (4.8)	0 (0)	0 (0)
II–III	0 (0)	0 (0)	0 (0)
III	0 (0)	0 (0)	0 (0)
Pulmonic valve regurgitation			
I	1 (4.8)	1 (11.1)	1 (16.7)
I–II	0 (0)	0 (0)	0 (0)
II	0 (0)	0 (0)	1 (16.7)
II–III	0 (0)	0 (0)	0 (0)
III	0 (0)	0 (0)	0 (0)
Medical History, n (%)			
Hypertension	20 (95.2)	9 (100)	5 (83.3)
Diabetes mellitus	10 (47.6)	2 (22.2)	1 (16.7)
Coronary heart disease	16 (76.2)	8 (88.9)	4 (66.7)
DCM	1 (4.8)	0 (0)	1 (16.7)
Concomitant medication, n (%)			
ACE-inhibitors	7 (33.3)	3 (33.3)	1 (16.7)
AT_1_-antagonists	9 (42.9)	1 (11.1)	1 (16.7)
Valsartan + sacubitril	4 (19.0)	1 (11.1)	1 (16.7)
Statins	19 (90.5)	7 (77.8)	3 (50.0)
Digitalis glycosides	0 (0)	1 (11.1)	1 (16.7)
β-blockers	12 (57.1)	7 (77.8)	6 (100)
SGLT2-inihibitors	4 (19)	1 (11.1)	1 (16.7)
Class IIII AADs	0 (0)	1 (11.1)	0 (0)

*AAD* antiarrthythmic drugs; *ACE* angiotensin-converting enzyme; *AT1* angiotensin II receptor type 1; *BMI* body mass index; *cAF* chronic atrial fibrillation; *DCM* dilated cardiomyopathy; *LVEF* left ventricular ejection fraction; *pAF* paroxysmal atrial fibrillation; *SGLT2* sodium-glucose linked transporter 2; *SR* sinus rhythm. *p < 0.05 versus SR; from ANOVA followed by Tukey multiple comparisons procedure for continuous variables and from Fisher’s exact test for categorical variables

To further transfer the results obtained in single CMs to the situation in a cellular composite and assess heart chamber specific differences, monolayers of atrial- and ventricular-like hiPSC-CMs were cultured in multi-well MEA plates (Fig. 2d). Consistent with the effects observed at the single CM level, dapagliflozin reduced the spike amplitude and spike slope of spontaneous field potentials (FPs) in a concentration-dependent manner under physiological temperature conditions (Fig. 2e, f). Interestingly, dapagliflozin effects were pronounced in atrial hiPSC-CMs compared to ventricular cells. For atrial hiPSC-CMs, administration of 30 µmol/L dapagliflozin reduced the spike amplitude to 48.2 ± 7.3% and the spike slope to 18.6 ± 6.0% of baseline values (Fig. 2e, f *left*). In contrast, no significant changes were observed in ventricular hiPSC-CM at a dapagliflozin concentration of 30 µmol/L (Fig. 2e, f *right*).

In conclusion, acute dapagliflozin treatment significantly lowered the excitability and affected fast depolarization in human CMs at concentrations in the low double-digit range, and effects were more pronounced in atrial compared to ventricular cells.

### ***Dapagliflozin decreases peak sodium currents in human atrial CMs by inhibiting Na***_***V***_***1.5 currents***

**Fig. 3:**
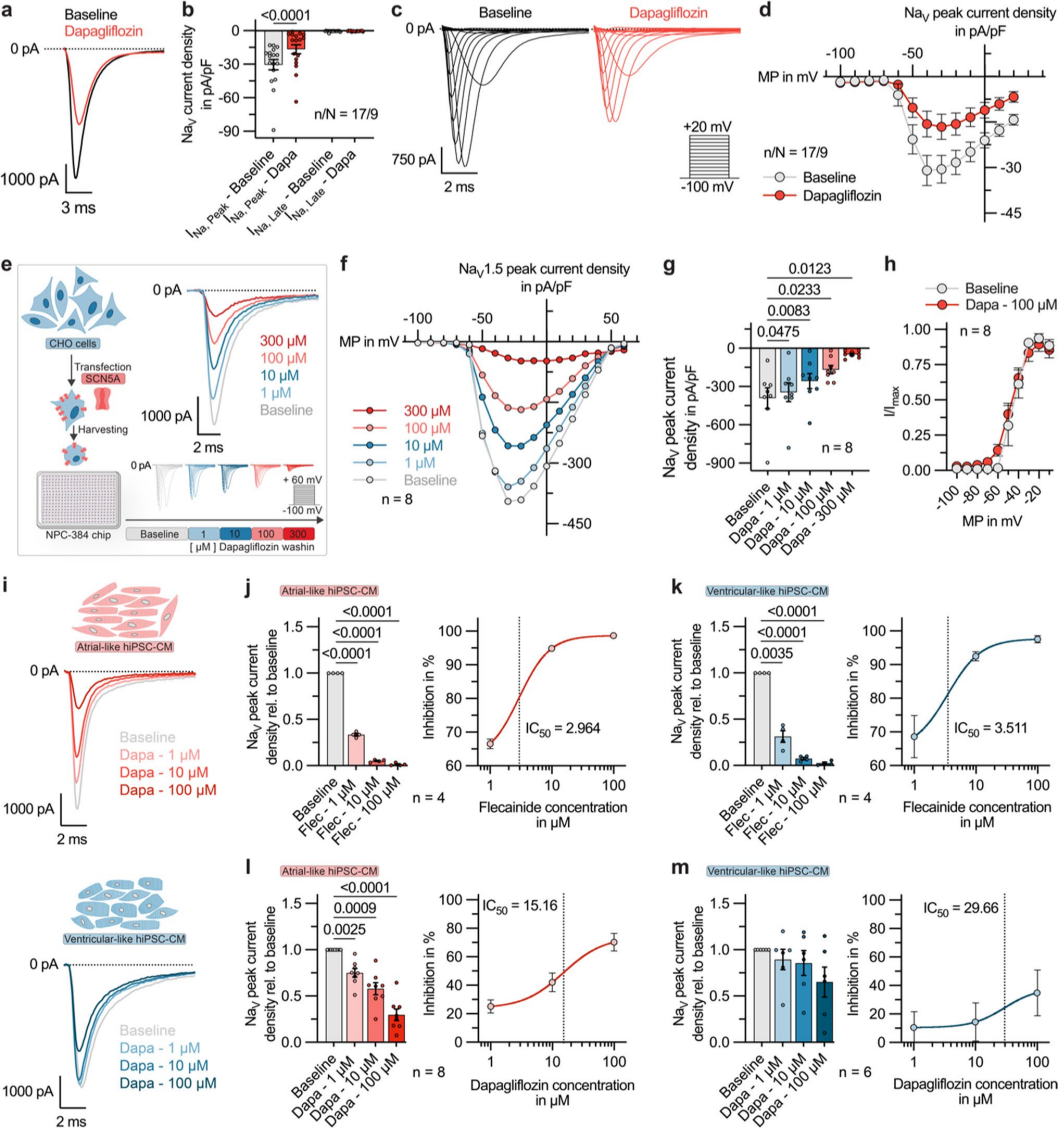
Dapagliflozin effects on sodium currents in human cardiomyocytes (CMs) and human Na_V_1.5 channels. **a** Representative recordings of peak sodium currents at − 30 mV, obtained from a human atrial CM at baseline and after dapagliflozin (100 µmol/L) treatment. **b** Na_V_ peak and late sodium current densities at − 30 mV, measured under baseline conditions and 5 min after administration of dapagliflozin (100 µmol/L; n/N = 17/9). P-values were derived from paired Student’s t-tests. **c** Representative families of sodium current traces, recorded from a human atrial CM under baseline conditions and 5 min after application of dapagliflozin (100 µmol/L). The voltage protocol is depicted as inset, **d** Current–voltage-relationship of Na_V_ peak current densities in human atrial CM before and 5 min after application of 100 µmol/L dapagliflozin (n/N = 17/9; MP, membrane potential). **e** *Left:* Experimental protocol: Transiently transfected Chinese hamster ovary (CHO) cells heterologously expressing human Na_V_1.5 channels were plated on an NPC-384 chip. Sodium currents were recorded using the SyncroPatch 384 Automated Patch Clamp (APC) system, under baseline conditions and while exposing the cells to increasing concentrations of dapagliflozin. *Right:* Representative Na_V_1.5 current traces, recorded with APC from transiently transfected CHO cells. **f** Current–voltage-relationship of Na_V_1.5 peak current densities at baseline and after stepwise increase of the dapagliflozin concentration from 1 to 300 µmol/L (n = 8). **g** Na_V_1.5 peak current densities of the respective recordings, quantified at -20 mV (n = 8). **h** Activation curve of Na_V_1.5 channels expressed in CHO cells calculated from Boltzmann fits under baseline conditions and dapagliflozin treatment (100 µmol/L; n = 8). **i** Representative sodium current traces, recorded with APC from atrial- and ventricular-like hiPSC-CM under baseline conditions and after administration of increasing dapagliflozin concentrations (1, 10, 100 µmol/L). **j** *Left:* Na_V_ peak sodium current densities relative to baseline values, recorded from atrial-like hiPSC-CM under baseline conditions and during perfusion with flecainide at increasing concentrations (1, 10, 100 µmol/L; n = 4). *Right:* Dose–response-curve of Na_V_ peak current inhibition by flecainide in atrial-like hiPSC-CM (IC_50_ = 2.96; n = 4). **k** *Left:* Na_V_ peak sodium current densities relative to baseline values, recorded from ventricular-like hiPSC-CM under baseline conditions and during perfusion with flecainide at increasing concentrations (1, 10, 100 µmol/L; n = 4). *Right:* Dose–response-curve of Na_V_ peak current inhibition by flecainide in ventricular-like hiPSC-CM (IC_50_ = 3.51; n = 4). **l** *Left:* Na_V_ peak sodium current densities relative to baseline values, recorded from atrial-like hiPSC-CM under baseline conditions and during perfusion with dapagliflozin at increasing concentrations (1, 10, 100 µmol/L; n = 8). *Right:* Dose–response-curve of Na_V_ peak current inhibition by dapagliflozin in atrial-like hiPSC-CM (IC_50_ = 15.16; n = 8). **m** *Left:* Na_V_ peak sodium current densities relative to baseline values, recorded from ventricular-like hiPSC-CM under baseline conditions and during perfusion with dapagliflozin at increasing concentrations (1, 10, 100 µmol/L; n = 6). *Right:* Dose–response-curve of Na_V_ peak current inhibition by dapagliflozin in ventricular-like hiPSC-CM (IC_50_ = 29.66; n = 6). Unless stated otherwise, data are given as mean ± SEM and P-values were derived from ordinary one-way analysis of variance (ANOVA). Where indicated, the peak sodium current amplitudes were normalized to the respective cell capacitance to obtain current densities

To gain a deeper understanding of the described effects on AP and FP formation, we first investigated the susceptibility of voltage-dependent sodium currents, carrying the upstroke of the cardiac action potential, to dapagliflozin. For this purpose, fast inward sodium currents were recorded in voltage-clamp experiments from isolated human atrial CMs. As shown in Fig. 3a dapagliflozin (100 µmol/L) caused a marked inhibition of voltage-dependent peak sodium current, which is consistent with the reduction of the CM’s excitability, APA and AP upstroke velocity we described before. Upon administration of dapagliflozin (100 µmol/L) peak sodium current densities at a membrane potential (MP) of -30 mV were significantly reduced by 53.6 ± 6.9% (Fig. 3b). In our experimental setup, we could only record very minor densities of the late sodium current component in unstimulated atrial cells, and therefore no significant changes between baseline and dapagliflozin were detected. We used the voltage-step protocol presented in Fig. 3c to investigate the voltage-current relationships before and after administration of dapagliflozin. As demonstrated in the representative current traces in Fig. 3c dapagliflozin inhibits peak sodium currents at a wide range of MPs (Fig. 3d).

Hypothesizing that dapagliflozin might suppress AP formation by inhibition of fast inward sodium currents, human Na_V_1.5 channels were heterologously overexpressed in CHO cells and subjected to functional characterization using the Nanion SyncroPatch 384 APC system (Fig. 3e). Following transient transfection, we elicited Na_V_1.5 currents using the voltage-step protocol depicted in Fig. 3e. Measurements performed under baseline conditions and during gradual administration of increasing concentration levels of dapagliflozin (1–300 µmol/L) confirmed a concentration-dependent inhibition of Na_V_1.5 peak current densities (Fig. 3e, f, g). At a concentration of 100 µmol/L dapagliflozin inhibited the peak current densities measured at -20 mV by 57.6 ± 6.1%. This is very consistent with the Na_V_ peak current reduction we found for human atrial CMs. Notably, the Na_V_1.5 peak current density was already significantly reduced at clinically relevant concentrations of 1 and 10 µmol/L dapagliflozin in transfected CHO cells (Fig. 3g). Dapagliflozin did not affect the half-activation potential of heterologously expressed Na_V_1.5 channels (Fig. 3h).

To further confirm and characterize a direct class I antiarrhythmic effect of dapagliflozin, we measured voltage-dependent sodium currents in atrial- and ventricular-like hiPSC-CM using the APC system and compared effects of flecainide and dapagliflozin on peak sodium current densities (Fig. 3i–m). Flecainide strongly inhibited peak sodium current densities at 1, 10, and 100 µmol/L in both atrial and ventricular hiPSC-CM, with IC_50_ values of 2.96 µmol/L and 3.51 µmol/L, respectively (Fig. 3j, k). In atrial hiPSC-CMs, dapagliflozin also led to a significant decrease in peak sodium current densities at 1, 10, and 100 µmol/L (Fig. 3l). The reduction was consistent with the reduction of peak current densities in CHO cells expressing Na_V_1.5 and in native human atrial CMs. The IC_50_ value for the inhibition of peak sodium currents in atrial hiPSC-CMs by dapagliflozin was 15.16 µmol/L, which is only about fivefold the IC_50_ value for flecainide (Fig. 3l). In ventricular-like hiPSC-CMs, the dapagliflozin effect on peak sodium current densities was attenuated compared to the atrial cells and the IC_50_ value was higher (Fig. 3m). To conclude, we first demonstrated that dapagliflozin inhibits peak sodium currents in native human atrial CMs. Secondly, we employed CHO cells expressing human Na_V_1.5 and hiPSC-CMs to further characterize this inhibition, and our findings showed that the inhibitory effects are comparable in all three settings and relevant at concentrations ranging from 1 to 100 µmol/L. This is in line with the differences in dapagliflozin effects on spike amplitude and spike slope we observed before.

Besides inward sodium currents, potassium currents like the transient outward potassium current (*I*_to_) and the ultrarapid outward potassium current (*I*_Kur_) could potentially contribute to the corresponding changes in AP formation. Therefore, we directly compared dapagliflozin effects on human potassium and sodium channels in a heterologous expression system (Fig. S5a–c). Here, the inhibitory effect of dapagliflozin on Na_V_1.5 was significantly stronger than effects of the SGLT2i on K_V_1.4, K_V_4.3, and K_V_1.5 channels (Fig. S5c). These findings were further confirmed by potassium current recordings on human atrial CMs (Fig. S5d–f). Dapagliflozin (1, 10, 100 µmol/L) did not significantly alter peak and late outward potassium current densities (Fig. S5e). The *I*_to_ component separated by a double pulse protocol by exploiting fast recovery from inactivation of *I*_to_ while *I*_Kur_ is still inactivated [10] was also not significantly changed under increasing concentrations of dapagliflozin (1, 10, 100 µmol/L) (Fig. S5f). However, we observed a non-significant trend towards moderate inhibition of *I*_to_ (26.6%), which is in line with a moderate inhibition of K_V_4.3 channels in the heterologous expression system (20.3%) (Fig. S5c, f).

In summary, acute dapagliflozin treatment inhibits Na_V_ peak current densities. The effect is comparable to the inhibition of fast inward sodium currents by the class I antiarrhythmic flecainide and pronounced in atrial cells. Additionally, dapagliflozin tends to moderately inhibit *I*_to_ currents.

### ***Inhibition of peak sodium currents might be mediated by direct binding of dapagliflozin to the Na***_***V***_***1.5 channel pore***

**Fig. 4:**
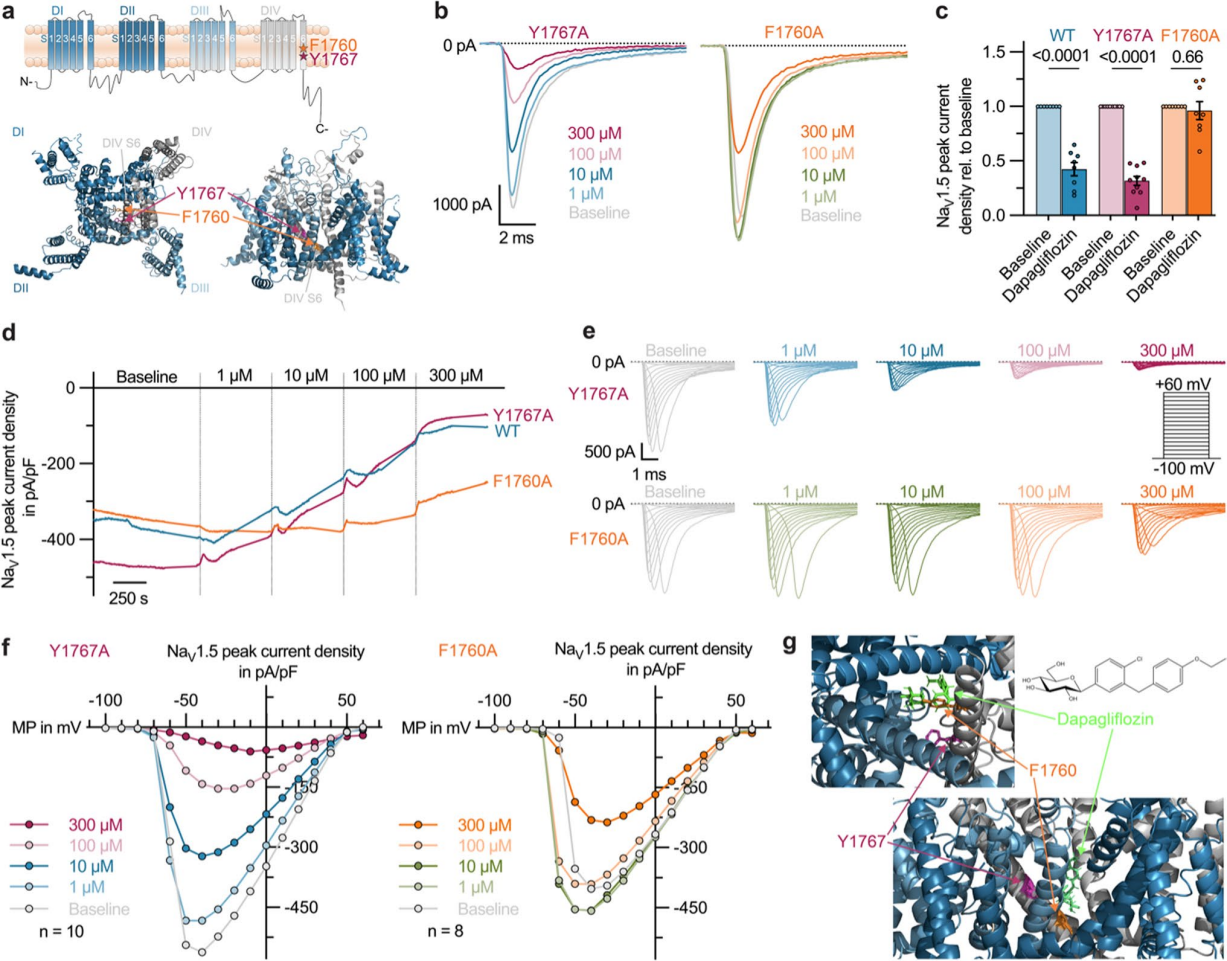
Investigation of the susceptibility of molecular drug binding sites of Na_V_1.5 to dapagliflozin. **a** Three-dimensional visualization of the Na_V_1.5 channel based on the recently revealed cryo-EM structure of the rat Na_V_1.5 ortholog (PDB ID: 6UZ0 [18]). The four repetitive domains of this pseudo-tetramer, each harboring 6 segments (S1–6) are visualized in different shades of blue and the pore-lining mutants F1760 and Y1767 located in the S6 segment of the fourth domain (DIV S6) are highlighted in orange and purple, respectively. **b** Representative Na_V_1.5 current traces, recorded with Automated Patch Clamp (APC) from Chinese hamster ovary (CHO) cells transiently transfected with *SCN5A* pore mutants Y1767A (*left*) and F1760A (*right*) during a gradual increase of the dapagliflozin concentration (1–300 µmol/L). **c** Na_V_1.5 peak current densities relative to baseline values, measured with APC in CHO cells heterologously expressing *SCN5A* wild-type (WT) or pore mutants Y1767A and F1760A at -20 mV under baseline conditions and after administration of dapagliflozin (100 µmol/L; n = 8–10). **d** Time course of Na_V_1.5 peak current densities, recorded with APC from CHO cells transfected with *SCN5A* WT, Y1767A or F1760A during gradual increase of the dapagliflozin concentration (1–300 µmol/L). **e** Representative families of sodium current traces, recorded with APC from CHO cells transfected with *SCN5A* Y1767A or F1760A during stepwise increase of dapagliflozin concentration (1–300 µmol/L). The pulse protocol is depicted as inset. **f** Current–voltage-relationship of Na_V_1.5 peak current densities, recorded with APC from CHO cells transfected with *SCN5A* Y1767A (*left*) or F1760A (*right*) at baseline and after stepwise administration of dapagliflozin (1–300 µmol/L) (n = 8–10; MP, membrane potential). **g** Hypothetical docking of dapagliflozin into the Na_V_1.5 channel pore. The regions of the excerpts are indicated in **(a)** as dashed squares. Data are provided as mean ± SEM and P-values were derived from paired Student’s t-tests

Theoretically, the inhibitory effect of dapagliflozin on Na_V_1.5 channels could be mediated either by interaction with one of the numerous signaling cascades regulating the channel [21, 47], or by direct binding of the drug into the channel pore. To investigate the molecular mode of action, we generated the two pore mutants *SCN5A*-Y1767A and *SCN5A*-F1760A. Therefore, aromatic amino acids known to contribute to the molecular drug binding site of the Na_V_1.5 channel were replaced by an alanine with a less reactive aliphatic residue. Located in the S6 segment of the fourth domain (DIV S6), these amino acid residues line the intracellular side of the Na_V_1.5 channel pore (Fig. 4a, visualization based on the cryo-EM structure of the rat Na_V_1.5 ortholog recently revealed by Jiang et al*.* 2020; PDB ID: 6UZ0) [18]. CHO cells, transiently transfected with *SCN5A*-WT, *SCN5A*-Y1767A, and *SCN5A*-F1760A, were subjected to APC measurements of peak sodium currents under baseline conditions and after application of 1–300 µmol/L dapagliflozin (Fig. 4b–f and Fig. S6a, b). Whereas for the *SCN5A*-Y1767A mutant inhibitory effects were similar to dapagliflozin effects observed for the WT channel, the *SCN5A*-F1760A mutant showed a significant decrease in the affinity of dapagliflozin to Na_V_1.5. Under 100 µmol/L dapagliflozin the Na_V_1.5 peak current density was reduced by 68.3 ± 4.2% in cells transfected with *SCN5A*-Y1767A, while there was no significant change in cells expressing the *SCN5A*-F1760A mutant (Fig. 4c). Further, comparing the wash-in curves of the two pore-lining mutants with WT channels (Fig. 4d) and analyzing the voltage-current relationships (Fig. 4e,f) strongly emphasizes that upon exchange of the pore-lining amino acid residue phenylalanine 1760, the affinity of dapagliflozin to Na_V_1.5 is significantly attenuated. We therefore suggest that the SGLT2i binds directly into the channel pore of Na_V_1.5 and that the amino acid phenylalanine 1760 contributes to the molecular drug binding site of dapagliflozin. Finally, in silico docking simulations of dapagliflozin into the intracellular pore of the aforementioned cryo-EM structure predicted a hypothetical binding site in close proximity to phenylalanine 1760 (Fig. 4g).

### Regulation of atrial sodium currents in chronic AF patients

**Fig. 5:**
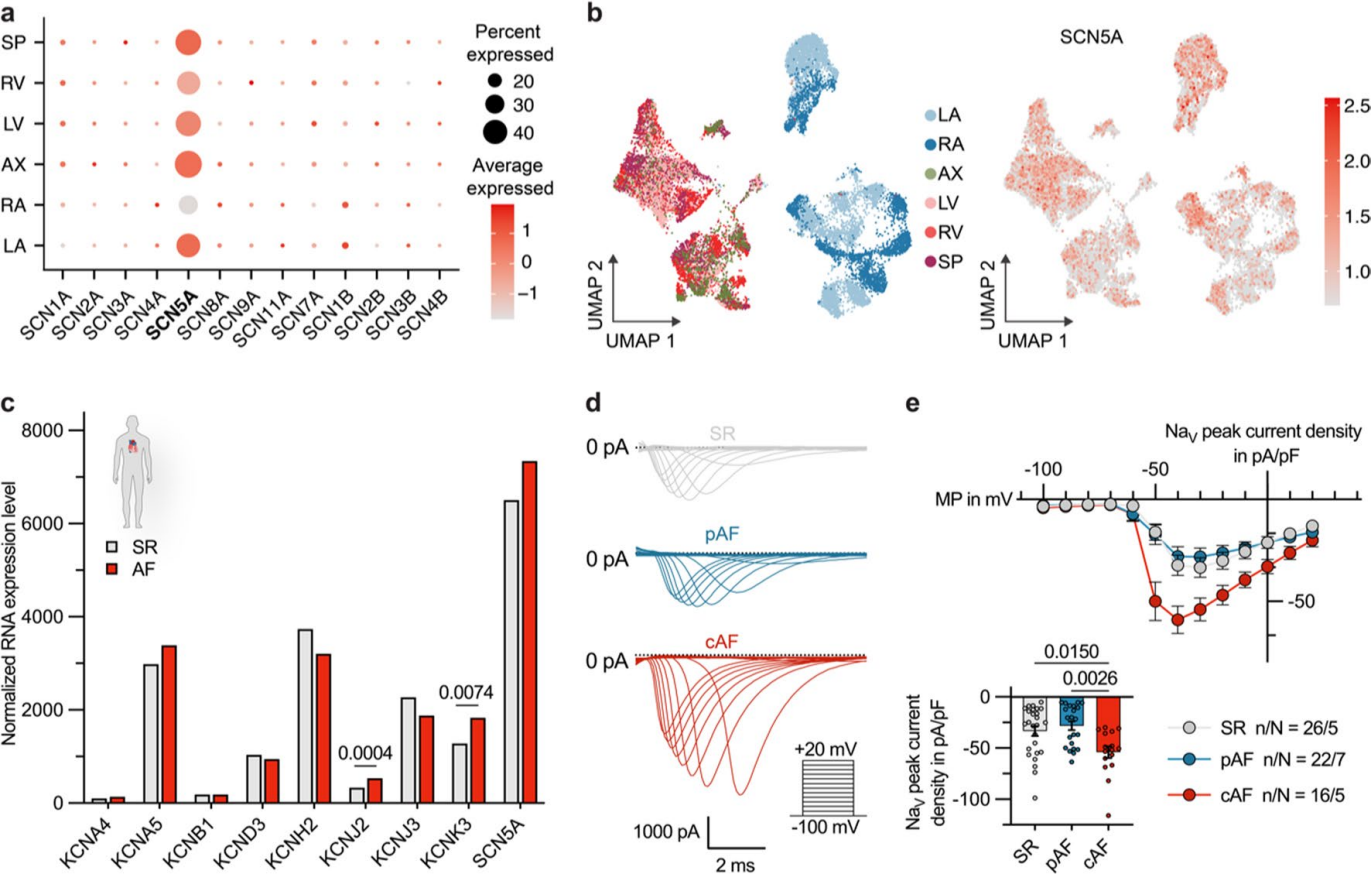
Differential sodium channel expression between cardiac regions and rhythm states. **a** Expression analysis of Na_V_ channel subunits in human cardiomyocytes (CM), based on data from the Heart Cell Atlas [23]. The size of the dots indicates the percentage of cells expressing the channel subunit within a cardiac region (AX, apex; LA, left atrium; LV, left ventricle; RA, right atrium; RV, right ventricle; SP, septum). The color represents the scaled average expression level across all cells within a cardiac sub compartment. **b** Uniform manifold approximation and projection (UMAP) embedding, highlighting cardiac regions on the left and normalized *SCN5A* expression on the right side. **c** Normalized RNA expression levels of important atrial ion channels compared between sinus rhythm (SR) and atrial fibrillation (AF) patients (DEseq2 analysis of bulk RNA sequencing data, derived from n = 15 SR vs. n = 15 AF samples). **d** Representative sodium current traces, recorded from atrial CMs of SR controls and patients with paroxysmal (pAF) or chronic (cAF) AF. **e** Na_V_ peak current densities quantified at a membrane potential (MP) of − 30 mV and current–voltage-relationships n atrial CMs obtained from N patients with SR, pAF or cAF (SR: n/N = 26/5, pAF: n/N = 22/7, cAF: n/N = 16/5). Data are shown as mean ± SEM and P-values were derived from ordinary one-way analysis of variance (ANOVA)

An analysis of a previously published single cell RNA sequencing dataset showed that the Na_V_1.5 channel encoded by the *SCN5A* gene is ubiquitously present in cardiomyocytes of all cardiac subcompartments, however *SCN5A* expression tends to be enhanced in ventricular compared to atrial cardiomyocytes (Fig. 5a, b).

Analysis of transcriptomic data obtained from poly(A)-enriched bulk RNA sequencing of right atrial tissue samples of n = 15 SR and n = 15 AF patients revealed, in addition to known characteristics of atrial electrical remodeling such as upregulation of *KCNJ2* and *KCNK3* expression [41, 42], a trend towards increased atrial *SCN5A* mRNA levels in AF samples (Fig. 5c). This observation is consistent with a significantly increased peak sodium current density in isolated atrial cardiomyocytes from cAF patients as compared to SR controls (peak sodium current densities at − 30 mV: SR: − 32.8 ± 5.3 pA/pF vs. pAF: − 28.3 ± 4.3 pA/pF vs. cAF: − 55.8 ± 7.4 pA/pF; Fig. 5d, e).

These data suggest that the inhibitory effect of dapagliflozin on sodium currents would be preserved in SR conditions and in the presence of atrial electrical remodeling, and might even counteract AF-associated changes in sodium channel function.

### Acute dapagliflozin treatment decreases atrial conduction velocity and can efficiently terminate AF episodes

**Fig. 6:**
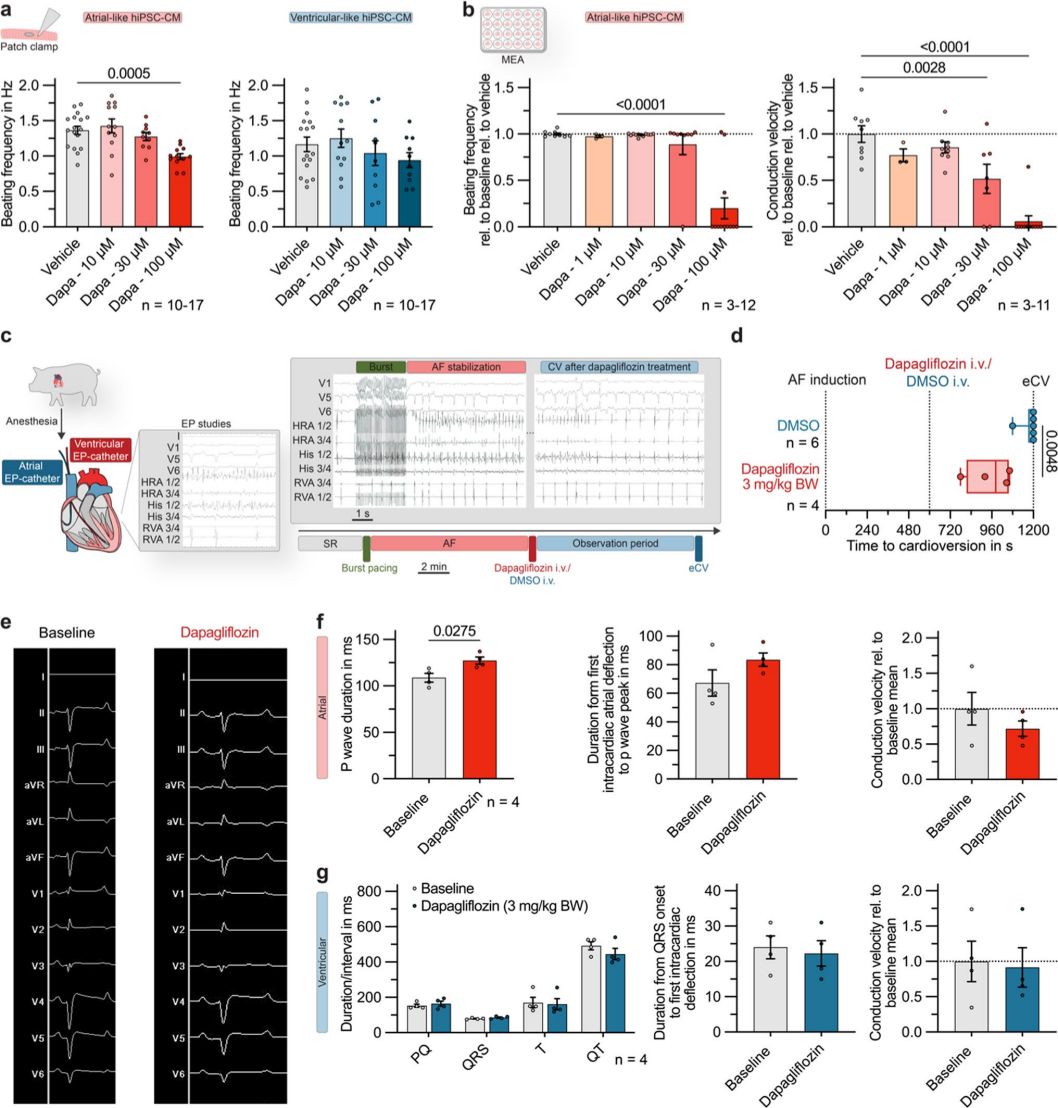
Antiarrhythmic effect of dapagliflozin in an in vivo large animal model of acute atrial fibrillation (AF). **a** Spontaneous beating frequency of atrial- and ventricular-like hiPSC-derived cardiomyocytes (hiPSC-CM), recorded with patch clamp under control conditions or exposition to dapagliflozin (10, 30, 100 µmol/L; atrial: n = 10–17; ventricular: n = 10–17). **b** Beating frequency (n = 3–12) and conduction velocity (n = 3–11) quantified from multi-electrode array (MEA) recordings of atrial-like hiPSC-CM monolayers relative to baseline values 15 min after application of dapagliflozin (1, 10, 30, 100 µmol/L) or the vehicle control. **c** Experimental protocol of a translational pilot study: Intracardiac electrophysiology (EP) catheters were inserted in anesthetized pigs and AF episodes were induced via atrial burst stimulation. If the AF episodes were stable within a 10 min control period, intravenous bolus administration of dapagliflozin (3 mg/kg body weight) or the appropriate solvent control (DMSO; dimethyl sulfoxide) was performed and the time to conversion to sinus rhythm (SR) was determined. If no conversion had occurred within 10 min, electrical cardioversion (eCV) was performed. *Grey box:* Representative EP recordings during burst stimulation and AF stabilization (*left*) and after administration of dapagliflozin (3 mg/kg body weight) showing cardioversion from AF to SR (*right*). **d** Conversion time in pigs treated with dapagliflozin (3 mg/kg body weight; n = 4) or the solvent control (n = 6). Box plots show the median and the interquartile range with whiskers ranging from minimum to maximum. The P-value was derived from Mann-Whitney-U-test. **e** Representative ECG recordings, obtained from pigs under control conditions and after treatment with dapagliflozin (3 mg/kg body weight). **f** Atrial parameters: P wave duration, duration from first intracardiac deflection to P wave peak and atrial conduction velocity in pigs under baseline conditions and after acute dapagliflozin treatment (3 mg/kg body weight; n = 4). **g** Ventricular parameters: ECG parameters, duration from QRS onset to first intracardiac deflection and ventricular conduction velocity in pigs under baseline conditions and after acute dapagliflozin treatment (3 mg/kg body weight; n = 4). Unless indicated otherwise, data are given as mean ± SEM and P-values were derived from Student´s t-tests

After investigating the molecular mechanism of Na_V_1.5 inhibition by dapagliflozin in depth, we wanted to assess the translational potential of the observed antiarrhythmic effects. In whole-cell patch clamp recordings of the spontaneous activity of single hiPSC-CMs, administration of dapagliflozin resulted in a concentration-dependent reduction in beating frequency. The effect was again more pronounced in atrial-like cells (Fig. 6a) and could further be confirmed for hiPSC-CM monolayers (Fig. 6b). Moving from the single cell to the cellular network level, in monolayers of atrial-like hiPSC-CM the conduction velocity was significantly decreased after administration of dapagliflozin at 30 µmol/L and 100 µmol/L concentrations (Fig. 6b).

To evaluate the acute antiarrhythmic potential of dapagliflozin strongly suggested by our in vitro studies, we used an established translational large-animal model of burst pacing-induced acute AF in vivo [40, 52–54]. In anesthetized pigs, AF episodes were induced by atrial burst-pacing via transjugular inserted electrophysiology (EP) catheters (Fig. 6c). If the AF episodes were stable within a 10 min control period, intravenous bolus administration of elevated-dose dapagliflozin (3 mg/kg body weight) or DMSO as appropriate solvent control at equal solvent concentrations was performed and the time to conversion to SR was determined. If no conversion occurred within 10 min, electrical cardioversion (eCV) was performed (Fig. 6c). Under acute dapagliflozin treatment a 100% conversion rate could be documented, as opposed to 16.7% under control treatment. The mean conversion times upon increased single-dose dapagliflozin treatment (5:52 ± 1.06 min) were significantly shorter in comparison with the solvent controls (Fig. 6d).

With regard to surface ECG parameters (Fig. 6e), animals acutely treated with dapagliflozin showed a significant prolongation of P-wave duration from 108.8 ± 4.8 ms to 127.3 ± 3.8 ms (Fig. 6f). Conversely, PQ interval, QRS duration, T-wave duration and QT intervals showed no significant changes (Fig. 6g). Interestingly, both the time interval from first deflection of the intracardiac atrial signal to the peak of the P-wave and the atrial conduction velocity determined over the two pairs of electrodes of the intracardiac catheter showed a clear trend toward reduction of atrial conduction velocity (Fig. 6f). In contrast, the time interval from the onset of the Q-wave on the surface ECG to the first deflection in the ventricular intracardiac catheter and the ventricular conduction velocity were virtually unchanged (Fig. 6g).

Concluding, the comprehensive investigations of direct electrophysiological dapagliflozin effects in different cellular as well as in vivo models strongly indicate that the SGLT2i could function as an acute antiarrhythmic agent lowering atrial excitability by direct inhibition of fast inward sodium currents (Fig. S7).

### Effects of sustained elevated-dose dapagliflozin treatment in a porcine model of persistent AF

**Fig. 7:**
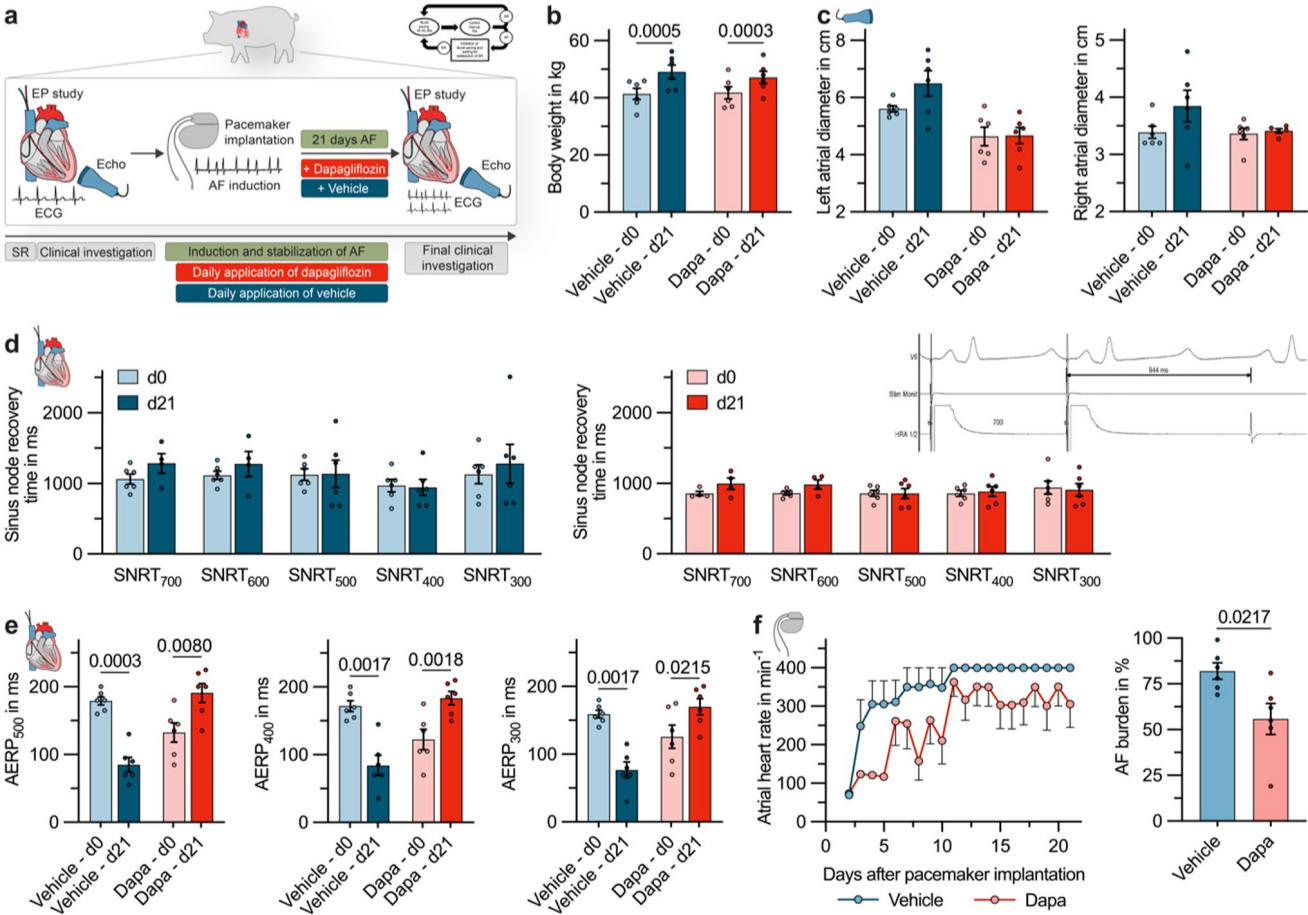
Elevated-dose dapagliflozin treatment for rhythm control in a translation large animal model of persistent atrial fibrillation (AF). **a** Experimental protocol: Following ECG, echocardiography, and electrophysiological (EP) studies, AV nodal ablation and dual-chamber pacemaker implantation were performed in pigs. AF was induced in n = 12 pigs over a three-week period. The pigs were randomized to dapagliflozin (3 mg/kg body weight/day *i.v.*; dapa; n = 6) or the corresponding solvent control (vehicle; n = 6). Finally, echocardiography and EP studies were repeated and the AF-burden over the three-week period was quantified by daily surface ECGs and pacemaker interrogation. The pacing protocol used to induce AF is shown as an inset. **b** Comparison of body weight between the respective groups of animals at the beginning and at the end of the experiment. **c** Left and right atrial diameter measured at day 0 and day 21 via echocardiography. **d** Sinus node recovery times (SNRTs) following 30 s of overdrive suppression by right atrial stimulation at basic cycle lengths from 300 to 700 ms, measured at day 0 and day 21 (n = 4–6, the dropouts result from measurements where the spontaneous cycle length of the pig was smaller than the respective basic cycle length). **e** Atrial effective refractory periods (AERPs) measured at a S1 cycle length of 500, 400, and 300 ms. **f** Mean atrial heart rates, derived from daily 6-lead surface ECGs. **g** AF load, quantified by the pacemaker devices as the time the animal spent in AF divided by the duration of the experiment. Unless indicated otherwise, data are given as mean ± SEM and P-values were derived from Student´s t-tests

To study the efficacy of long term dapagliflozin treatment in rhythm control, dapagliflozin was administered daily in a porcine model of persistent AF [40, 52–54]. In all animals, echocardiography and invasive EP studies were conducted (Fig. 7a), a dual chamber pacemaker was implanted, and an AV node ablation was performed to prevent the development of tachycardia-induced heart failure as a consequence of AF. Right ventricular backup pacing was provided via the implanted pacemakers. Subsequently, 12 pigs were randomized to an AF-induction group receiving daily treatment with dapagliflozin (3 mg/kg body weight/day *i.v.*; n = 6) or an AF-induction group treated with the respective vehicle (n = 6) (Fig. 7a). AF was induced by right-atrial burst stimulation, using a feedback-algorithm which allows for endogenous propagation of AF [40, 52–54]. During the follow-up period of 21 days, atrial burst stimulation was alternated with pacing-free intervals for automatic rhythm assessment by the pacemakers. At the end of the observation period, echocardiography and EP studies were repeated. Animals on daily high-dose dapagliflozin therapy had a similar weight gain compared with the control group (Fig. 7b). While a significant increase of right and left atrial diameters was noted in the control group, this could be entirely reversed by dapagliflozin treatment (Fig. 7c). Dapagliflozin did not significantly affect sinus node recovery times (SNRTs) measured after 30 s of overdrive suppression at different S1 cycle lengths (Fig. 7d). As expected, AF-induction was associated with a significant reduction of atrial effective refractory periods (AERPs) in pigs receiving control treatment. This was entirely reversed by treatment with dapagliflozin (Fig. 7e). Finally, after 3 weeks, animals under dapagliflozin therapy showed markedly reduced mean atrial heart rates, derived from daily surface ECG recordings and a significantly attenuated AF-burden (Fig. 7f).

**Fig. 8:**
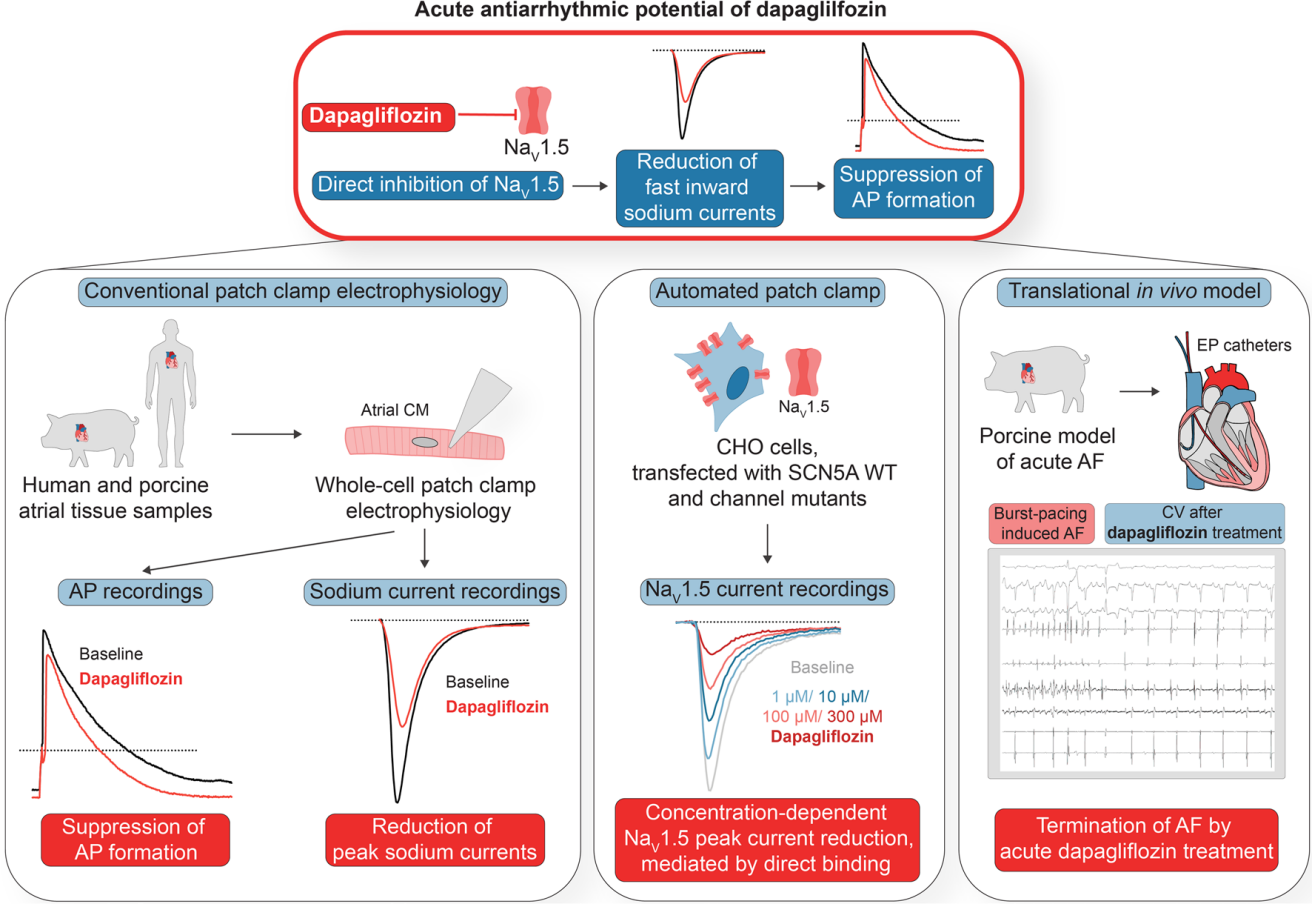
Graphical abstract: Acute antiarrhythmic potential of dapagliflozin

These experiments confirm that the direct electrophysiological effect of dapagliflozin we characterized on the cellular level indeed translates to antiarrhythmic effects in vivo that can be employed for rhythm control of persistent AF (as summarized in Fig. 8).

## Discussion

In this study, we analyzed acute effects of dapagliflozin on atrial electrophysiology, which resembled the action of a class I antiarrhythmic agent. In isolated native atrial CMs, we showed that acute application of increased single-dose dapagliflozin reduces AP inducibility, the APA and maximum upstroke velocity. We comprehensively characterized the inhibition of *I*_Na_ by direct binding of dapagliflozin to the Na_V_1.5 channel pore as the underlying molecular mechanism. *I*_Kur_ and *I*_to_, which could potentially also contribute to changes in APA, were not or only very moderately altered, respectively. In atrial and ventricular-like hiPSC-CMs, we confirmed the direct electrophysiological effect of dapagliflozin on both the single cell and the monolayer level, and additionally discovered atrial-predominance of the effects. Finally, dapagliflozin could be effectively employed for cardioversion of acute AF episodes and rhythm control of persistent AF in a translational large-animal model. Importantly, our results indicate a direct antiarrhythmic effect of elevated doses of dapagliflozin that could be beneficial in the treatment of atrial arrhythmias. The observed effects indicate a novel mechanism and suggest the further investigation of new indications for SGLT2i treatment. Acute transient increase in dapagliflozin doses may be a new option to address frequently occurring atrial arrhythmias in HF patients. At the same time, these results further promote the application of dapagliflozin as an acute antiarrhythmic agent in AF patients, regardless of HF.

The molecular mechanism underlying the acute antiarrhythmic effects of dapagliflozin we discovered and characterized in this study is the direct inhibition of peak sodium currents in human atrial CMs. Previously, it has been shown that empagliflozin, dapagliflozin and canagliflozin block the late component of voltage-gated cardiac Na^+^ channels (late-*I*_Na_) on ventricular CMs derived from a murine transverse aortic constriction (TAC)-induced pressure overload model [34]. In the TAC model, empagliflozin (10 µmol/L) reduced total and late sodium currents while peak currents were not affected [34]. The description of this mechanism contributed to the understanding of the cardioprotective effects of chronic SGLT2i treatment in patients with HF. In contrast, we aimed to go beyond the current indications for SGLT2 inhibitor treatment and place special emphasis on the atria to identify molecular evidence for antiarrhythmic effects of SGLT2 inhibitors, which has not been done before. In our work, the effect of dapagliflozin on atrial CMs has now been studied for the first time. Here, we observed a significant inhibition of peak sodium currents in human atrial CMs, while we did not detect relevant late sodium current densities in the atrial cells. This could be explained by the fact that, while Philippaert et al*.* utilized TAC in vivo or H_2_O_2_ in vitro for stimulation, we did not specifically induce late sodium currents. However, it ensures that, for the electrophysiological effects in our experimental setting, the peak current component is of main relevance. The significant reduction of the excitability of atrial CMs we observed upon acute application of elevated-dose dapagliflozin is a previously unrecognized mechanism and might advance SGLT2i treatment strategies, as patients could benefit from an individual combination of chronic low and acute increased-dose dapagliflozin treatments. Furthermore, while late sodium currents play an important role in the pathophysiology of HF, mechanisms involving the peak sodium current component might be of great interest also for patients with atrial arrhythmias but preserved ventricular function.

Na_V_1.5 channels are regulated by a variety of intracellular signaling cascades including the AMP-activated protein kinase [21] or CaMKII [47], which both have been described to be inhibited by SGLT2i. Philippaert et al*.* suggested that the inhibition of late sodium currents by SGLT2i is caused by a direct channel blockade in close proximity to the pore region, whereas, further studies pointed towards a potential role of CaMKII in mediating the inhibition [16, 34]. Our own experiments showed that exchange of aromatic amino acids at the inner pore of Na_V_1.5 channels dramatically decreased the interaction of dapagliflozin and Na_V_1.5. Therefore, we suggest that the peak sodium current reduction is more likely mediated by a direct interaction with the Na_V_1.5 pore, and that the binding site of dapagliflozin has an overlap with binding sites of flecainide and local anesthetics. However, it is very interesting that classical Na_V_1.5 blockers that interact with this site, such as several local anesthetics, show a rate-dependency of peak sodium inhibition that could not be observed for dapagliflozin, at least in the range of 0.5 to 2 Hz [34]. Concluding, there might be mechanistic parallels between the effects of dapagliflozin on Na_V_1.5 peak sodium currents that we observed and the previously described inhibition of late sodium currents by SGLT2i and patients could benefit from the specific combination of those effects. For example, both appear to be mediated by the same drug binding site within the Na_V_1.5 channel. Still, the question remains why Philippaert et al*.* did not observe changes in peak sodium currents at 10 µmol/L, while we found significant inhibition of peak sodium currents as well as the AP inducibility, APA, and upstroke velocity by 10 µmol/L dapagliflozin. Besides a potential role of differences in murine and human cardiac electrophysiology and disease states, this could be explained by differing sensitivity to the inhibitory SGLT2i effects in ventricular and atrial CMs.

To address this, we compared dapagliflozin effects on hiPSC differentiated in atrial- and ventricular-like CMs. Interestingly, both the inhibition of peak sodium currents measured with APC and the reduction in spike amplitude and slope of FPs recorded with MEA were pronounced in atrial hiPSC-CMs, compared to ventricular cells. “Atrial-selective” effects of sodium blocking agents like ranolazine and flecainide have been described before [1, 7, 35, 36]. The authors discussed that the increased atrial effectiveness might be explained by altered expression and/or biophysical properties of sodium channels in atrial and ventricular CMs. The atrial predominance of class I antiarrhythmic SGLT2i effects we showed in our study can account for deviation from prior findings in ventricular CMs, and constitutes a very important and interesting finding from a translational perspective.

The direct effects of dapagliflozin on the fast depolarization phase of APs/FPs were consistent in human and porcine native atrial CMs and hiPSC-CMs. For various class I antiarrhythmic agents it is known that, besides inhibitory effects on sodium currents, they can also influence potassium channels and APDs in distinct ways. For dapagliflozin, APD_50_ and APD_90_ values were shortened in porcine atrial CMs. However, in human atrial CMs acute dapagliflozin treatment led to a mild increase in APD_90_. In general, shortening of the AP in this context could be explained by inhibition of late sodium currents. Previously, it was shown that blockade of late *I*_Na_ by ranolazine leads to a shortening of APD in guinea pig CMs [2]. Additionally, less activation of* I*_Ca,L_ channels due to the smaller AP amplitude might also contribute to APD shortening. On the other hand, especially class Ia antiarrhythmic drugs are known to prolong the AP by inhibiting potassium channels. Therefore, the deviating effects of dapagliflozin on APDs in porcine and human CMs might be a result of differing late sodium and/or potassium current properties, and it will be interesting and necessary to further investigate effects of SGLT2i on other ion channels in the future.

In patients receiving a daily dose of 10 mg dapagliflozin or empagliflozin, plasma concentrations were reported to range between 0.4 and 1.1 μM [38, 39]. A plasma protein binding of 80–99% resulted in free plasma concentrations of 10 to 200 nM [9]. However, potential accumulation of dapagliflozin in lipophilic environments such as the plasma membrane over time in vivo is the subject of current debates [37]. To study antiarrhythmic drug effects, it is crucial to determine the acute effects of the substance on single cardiomyocyte electrophysiology. Although it is always difficult to extrapolate concentration–response curves from artificial in vitro models to in vivo conditions, the effects were clearly concentration-dependent and had their maximum effect at higher concentrations than those achieved with currently approved antidiabetic doses. Nevertheless, the measurements of dapagliflozin effects on human Na_V_1.5 channels presented in this work show that the SGLT2i exerts significant effects on peak sodium currents already in the one- to two-digit micromolar range. Furthermore, translational small animal models investigating the effect of different SGLT2i on cardiac function used doses in the range of 10–150 mg/kg per day, which exceed the clinically used daily doses of 10 mg dapagliflozin or 10 mg empagliflozin for a 50–100 kg patient by several orders of magnitude (Table S1). The results of this work suggest that a wide range of cardiac patients may benefit from the temporary application of higher than currently approved doses of SGLT2i, based on their acute antiarrhythmic mode of action. Generally, advantages of increased-dose treatment could also be true for patients with diabetes because exposure–response analyses suggest that maximum antidiabetic effects are not reached with the 10 mg dose [20]. In our study, the *i.v.* dapagliflozin doses used in vivo to efficiently terminate acute AF episodes was around 25–30 times higher than the normal *p.o.* dose of 10 mg. 50 times the currently recommended human dose has already been tested in healthy subjects in former studies and did not show any toxicity. No meaningful effect on electrolyte balance or the QT_c_ interval and no increase in the incidence of hypoglycemia compared to placebo could be observed [12]. Another early clinical safety study demonstrated that oral increased-dose administration of SGLT2i empagliflozin resulting in a maximum plasma concentration around 8 µmol/l was free of clinical side effects [45]. Still, the safety profile would have to be even further investigated in translational and clinical studies.

Because SGLT2 and Na_v_1.5 inhibition are distinct pharmacological properties, molecular optimization may provide opportunities to optimize the relationship between SGLT2 and Na_v_1.5 inhibitory properties of these compounds and ultimately allow assessing the importance of each mechanism to the beneficial cardiac effects of SGLT2i.

The impressive effect of dapagliflozin on AP formation on the single CM level translated to a decrease in conduction velocities in both monolayers of atrial hiPSC-CM in vitro as well as porcine atria in vivo. This acute decrease in atrial conduction velocity was sufficient to cardiovert acute AF episodes in pigs, which finally confirmed the acute antiarrhythmic potential of dapagliflozin. Building on this, we investigated effects of daily elevated dose dapagliflozin treatment in a translational porcine model of persistent AF. Here, found a significant decrease in the AF burden as well as structural and electrophysiological parameters for AF-associated atrial remodeling upon dapagliflozin treatment. Previously, it was shown in a rat model of mitral regurgitation-induced myocardial dysfunction that dapagliflozin not only improved cardiac hemodynamic but also reduced the inducibility and duration of pacing-induced AF episodes on excised hearts, while surface ECG parameters remained unchanged [3]. Moreover, there are data from a canine large animal model of burst pacing-induced AF suggesting that treatment with canagliflozin suppresses atrial remodeling. After three weeks of SGLT2i treatment, AERP reduction and conduction velocity decrease were less pronounced than in the control pacing group, accompanied by a reduction in AF inducibility [28]. Our study demonstrates both acute cardioversion of paroxysmal AF episodes and rhythm control in a translational model of persistent AF, and provides important mechanistical insights on the direct electrophysiological mode of action.

In T2DM and HF patients, a significant reduction of AF burden under treatment with SGLT2i could be shown not only in randomized trials and their meta-analyses [14, 57] but also in retrospective registry studies [8] and a large-scale analysis of a pharmacovigilance database [5]. However, our findings indicate that the class I antiarrhythmic effects of elevated dose dapagliflozin might also function independent of T2DM or HF. Therefore, large randomized controlled trials with atrial arrhythmias as primary endpoints are necessary to specifically assess which patient groups could benefit from the antiarrhythmic effects of SGLT2i and under which dosage a balanced compromise of antiarrhythmic effect and side effect profile could be achieved.

The presence of T2DM is associated with oxidative stress, insulin resistance, atherogenesis, and endothelial dysfunction, all of which favor structural, electrical, and autonomic remodeling processes that underlie atrial cardiomyopathy [19, 29, 51]. Although it has not been proven by randomized trials, it is therefore likely that treatment of hyperglycemia also reduces the risk of developing AF [29, 51]. However, the antiarrhythmic effect of SGLT2i seems to clearly go beyond its antidiabetic action as it is more pronounced compared to other antidiabetic drugs and is also present in patients without the diagnosis of T2DM [5, 22].

Subgroup analyses pointed out, that the benefit of empagliflozin described in the EMPA-REG OUTCOME trial was particularly pronounced in AF patients [4]. Interestingly, we also observed that the reduction of AP inducibility by dapagliflozin was more pronounced in CMs obtained from AF pigs compared to SR controls. This may indicate that the antiarrhythmic effect constitutes a particular driver of the pleiotropic SGLT2i effects in HF patients. Contrarily, it is well known that most class I antiarrhythmic drugs have negative inotropic effects [46]. Furthermore, in studies of patients with ischemic heart disease or systolic HF, the proarrhythmogenic potential of class I antiarrhythmic drugs was associated with an excess in mortality [13]. Therefore, future studies will have to investigate whether Na_V_1.5 inhibition by SGLT2i has a pro-arrhythmic effect on the ventricles or whether the additional pleiotropic ventricular effects of SGLT2i lead to other outcomes compared to conventional class I antiarrhythmic drugs. It might be possible that in HF patients, the immediate, temporary effect of increased-dose SGLT2i acting as class I antiarrhythmic drugs on the ventricles would be masked by perhaps stronger effects related to reverse remodeling in the myocardium by chronic low dose treatment, and that SGLT2i therefore would promote fewer detrimental ventricular arrhythmias than classical class I antiarrhythmics. In this context, it is again particularly interesting to mention that the dapagliflozin effects on sodium currents, APs, and conduction velocities were stronger in atrial cells/tissue, compared to the ventricles.

In summary, we have shown in several complementary models that increased-dose dapagliflozin decreases atrial CM excitability by inhibiting Na_V_1.5 peak currents. The resulting acute class I antiarrhythmic effects were sufficient to terminate induced AF episodes and achieve rhythm control in clinically relevant porcine large animal models of paroxysmal and persistent AF. Our results suggest that acute increased-dose dapagliflozin treatment might be a novel therapeutical option for the treatment of atrial arrhythmias. This lays the groundwork for a new indication for acute SGLT2i treatment as an additional improvement of the therapeutic strategy in HF patients and as a completely new option in the treatment of AF patients with preserved ventricular function. While the antiarrhythmic effects of SGLT2i need to be further validated in prospective clinical trials, SGLT2i should be prioritized in accordance with current guidelines for the therapeutic management.

### Supplementary Information

Below is the link to the electronic supplementary material.

Supplementary file1 (DOCX 19572 KB)

## Data Availability

All underlying raw data are available from the corresponding author upon reasonable request.

## Abbreviations

ACE: Angiotensin-converting enzyme
AERP: Atrial effective refractory period
AF: Atrial fibrillation
ANOVA: Analysis of variance
AP: Action potential
APA: Action potential amplitude
APC: Automated patch clamp
APD_50_: Action potential duration at 50% repolarization
APD_90_: Action potential duration at 90% repolarization
AT1: Angiotensin II receptor type 1
AX: Apex
BMI: Body mass index
cAF: Chronic atrial fibrillation
CHO: Chinese hamster ovary
CM: Cardiomyocyte
DCM: Dilated cardiomyopathy
DMSO: Dimethyl sulfoxide
eCV: Electrical cardioversion
EGTA: Ethylenebis (oxyethylenenitrilo) tetraacetic acid
EP: Electrophysiology
FP: Field potential
HF: Heart failure
hiPSC: Human induced pluripotent stem cell
hiPSC-CM: HiPSC-derived CM
LA: Left atrium
LV: Left ventricle
LVEF: Left ventricular ejection fraction
MEA: Multi-electrode array
MP: Membrane potential
pAF: Paroxysmal atrial fibrillation
RA: Right atrium
RMP: Resting membrane potential
RV: Right ventricle
SGLT2: Sodium-glucose linked transporter 2
SGLT2i: SGLT2 inhibitor
SNRT: Sinus node refractory period
SP: Septum
TAC: Transverse aortic constriction
T2DM: Type 2 diabetes mellitus
UMAP: Uniform manifold approximation and projection
WT: Wild-type
