# Supplementary material for: Acute antiarrhythmic effects of SGLT2 inhibitors–dapagliflozin lowers the excitability of atrial cardiomyocytes
Source: Basic Res Cardiol. 2024 Jan 3;119(1):93–112. doi: 10.1007/s00395-023-01022-0 (PMC10837223; doi:10.1007/s00395-023-01022-0)
Supplement: Supplementary file 1 — Supplementary file1 (DOCX 19572 KB) [file 395_2023_1022_MOESM1_ESM.docx]

Basic Research in Cardiology - Supplementary material:

Acute antiarrhythmic effects of SGLT2 inhibitors – dapagliflozin lowers the excitability of atrial cardiomyocytes

**Author names:** Amelie Paasche^1,2,3†^, Felix Wiedmann^1,2,3†^, Manuel Kraft^1,2,3^, Fitzwilliam Seibertz^4,5,6^, Valerie Herlt^1^, Pablo L. Blochberger^1^, Natasa Jávorszky^1^, Moritz Beck^1^, Leo Weirauch^1^, Timon Seeger^1,2^, Antje Blank^7^, Walter E. Haefeli^7^, Rawa Arif^8^, Anna L. Meyer^8^, Gregor Warnecke^8^, Matthias Karck^8^, Niels Voigt^4,5,6^, Norbert Frey^1,2,3^, Constanze Schmidt^1,2,3*^

**Affiliations:**

^1^Department of Cardiology, University Hospital Heidelberg; Im Neuenheimer Feld 410, 69120 Heidelberg, Germany.

^2^DZHK (German Center for Cardiovascular Research), partner site Heidelberg /Mannheim, University of Heidelberg; Im Neuenheimer Feld 669, 69120 Heidelberg, Germany.

^3^HCR, Heidelberg Center for Heart Rhythm Disorders, University Hospital Heidelberg; Im Neuenheimer Feld 410, 69120 Heidelberg, Germany.

^4^Institute of Pharmacology and Toxicology, University Medical Center Göttingen; Robert Koch Strasse 42a, 37075 Göttingen, Germany.

^5^DZHK (German Center for Cardiovascular Research), Partner Site Göttingen; Robert Koch Strasse 42a, 37075 Göttingen, Germany.

^6^Cluster of Excellence "Multiscale Bioimaging: from Molecular Machines to Networks of Excitable Cells" (MBExC), University of Göttingen; Robert Koch Strasse 40, 37075 Göttingen, Germany.

^7^Department of Clinical Pharmacology and Pharmacoepidemiology, University Hospital Heidelberg; Im Neuenheimer Feld 410, 69120 Heidelberg, Germany.

^8^Department of Cardiac Surgery, University Hospital Heidelberg; Im Neuenheimer Feld 410, 69120 Heidelberg, Germany.

*Corresponding author:

Prof. Dr. med. Constanze Schmidt, FESC, FEHRA

Department of Cardiology

Medical University Hospital Heidelberg

Im Neuenheimer Feld 410

D-69120 Heidelberg, Germany

Tel.: ++49 6221 5636779

Fax: ++49 6221 565724

E-Mail: Constanze.Schmidt@med.uni-heidelberg.de

†A.P. and F.W. contributed equally to this work.

**Supplementary materials and methods**

***Animal handling and porcine atrial fibrillation (AF) model***

Pigs were sedated with azaperone (Elanco, Bad Homburg, Germany), midazolam (Hameln Pharma Plus, Hameln, Germany), and ketamine (Zoetis Deutschland, Berlin, Germany) and anaesthetized with propofol (Fresenius Kabi, Bad Homburg, Germany). Buprenorphine (Bayer Vital Tiergesundheit, Leverkusen, Germany) was used for analgesia. After completion of echocardiographic and electrophysiological (EP) studies isoflurane (Baxter Deutschland GmbH, Heidelberg, Germany) was used to maintain the anaesthesia. Following AV-nodal (AVN) ablation and implantation of a dual-chamber pacemakers (Abbott Medical, Eschborn, Germany), AF was induced by atrial burst pacing episodes (30 s, 40 Hz) as described [17]. Heart rhythm was monitored by the pacemakers and burst pacing episodes were suspended as soon as endogenous AF was detected. Hereby, AF was maintained for up to 8 weeks before pigs were euthanized by *i.v.* injection of potassium chloride under deep anaesthesia and hearts were explanted during final surgery. To test the antiarrhythmic effects of dapagliflozin at the whole-organism level, a translational model of acute artificially induced AF episodes previously described in detail was used [14, 16, 17]. In brief: pigs were anesthetised as described above, two quadripolar electrophysiology catheters were inserted via cannulation of the jugular vein, and AF was induced via right-atrial burst stimulation (2–8 s bursts, at 400–1,200 min^-1^, 10 V, 2.9 ms duration). After initiation of AF episodes, the atrial rhythm was monitored for 10 min to assess the stability of AF. Pigs that remained in AF underwent *i.v.* treatment with dapagliflozin or the respective solvent and the time to conversion was monitored. If a pig remained in AF for more than 20 min, electrical cardioversion (eCV) was performed. No further pharmacological experiments were performed in one individual animal for at least 8 plasma half-lives of dapagliflozin. Furthermore, to investigate the potency of high-dose dapagliflozin for rhythm control of persistent AF, the model of right atrial burst pacing after ablation of the AVN described above was applied. The animals were further implanted with a central venous catheter tunnelled to the neck via which a daily *i.v.* application of dapagliflozin was administered during feeding. Dapagliflozin 3 mg/kg body weight was administered as intravenous bolus and freshly diluted from a DMSO stock (50 mg/mL).

Access to the jugular vein was obtained using Seldinger technique. Under fluoroscopic guidance, quadripolar catheters were positioned at the junction between the superior vena cava and the right atrium and in the apex of the right ventricle. Intracardiac stimulation was achieved using a UHS 20 stimulus generator (Biotronik, Berlin, Germany) and the Bard Electrophysiology Clearsign (Bard Electrophysiology Division, Lowell, MA, USA) was employed for recording, analysis, and storage of electrocardiograms. Pacing thresholds ranged from 0.5–3 V at 2.9 ms and stimulation was performed at twice the diastolic pacing threshold. Atrial effective refractory periods (AERP) were measured using a conditioning train of 9 basic stimuli (S1; 500 ms, 400 ms or 300 ms as indicated) followed by an extra stimulus (S2) starting at 70 ms. Coupling intervals of extra stimuli were increased in 5 ms decrements until capture of the S2 stimulus was achieved. To measure sinus node recovery time (SNRT), atrial simulation was applied at basic cycle lengths of 700–300 ms for 30 seconds and praeautomatic pauses from the last stimulus to the first intrinsic atrial activation were measured. Corrected sinus node recovery times (cSNRTs) were calculated by subtracting the intrinsic cycle length from the respective SNRT. Surface electrocardiograms (ECGs) were recorded using conventional adhesive electrodes (3M red dot, 3M, Maplewood, MN, USA) in the classical Einthoven /Goldberger /chest-lead configurations and QT-intervals were corrected using Bazett’s formula. Echocardiography was performed in anaesthetised and spontaneously breathing animals (Vivid E9, CE-Healthcare Chicago, IL, USA). Left and right atrial diameters were measured in a modified dual chamber view, obtained from parasternal acoustic windows.

Oocytes for the two-electrode voltage clamp measurements were harvested from *Xenopus laevis* (Xenopus Express, Vernassal, France). The frogs were kept according to the Directive 2010/63/EU of the European Parliament and all animal experiments were authorised by the local Animal Welfare Committee (Regierungspräsidium Karlsruhe, reference number G165-19). Tricaine (1 g/L, pH 7.5; Pharmaq, Fording bridge, United Kingdom) was used to anaesthetise the frogs before extraction of the ovarian lobes. The ovarian lobes were then separated into small groups and Collagenase D (Roche Diagnostics, Mannheim, Germany) was used to remove collagenous tissue. Oocytes were sorted out under optical control and kept in standard oocyte solution containing (in mmol/L) 100 NaCl, 2 KCl, 1 MgCl_2_, 1.8 CaCl_2_, 5 HEPES, 2.5 pyruvic acid, and 50 mg/L gentamicin sulphate, pH 7.7 (with NaOH). A maximum of four operations was performed on each frog before they were killed by decerebration and pithing.

***Cardiomyocyte (CM) isolation***

Fresh human and porcine cardiac tissue samples were kept in a Ca^2+^-free cardioplegic solution containing (in mmol/L) 50 NaCl, 50 KCl, 6 KH_2_PO_4_, 25 MgSO_4_, 250 taurine, 25 MOPS, 20 glucose and 30 2,3-butanedion monoxime (pH 7.0) at 4–8°C for transport. Samples were either flash-frozen in liquid nitrogen and stored at -80°C for further transcriptomic analysis or placed in cardioplegic solution to cut off connective and fatty tissue and prepare 1–2 mm^3^ large pieces for enzymatic digestion. The tissue was rinsed in ethylenebis(oxyethylenenitrilo)tetraacetic acid (EGTA)-containing solution (in mmol/L): 137 NaCl, 5 KH_2_PO_4_, 1 MgSO_4_, 5 HEPES, 10 glucose, 10 taurine, 0.2 EGTA; pH 7.4) for 5 min and samples were digested in EGTA- and Ca^2+^-free solution containing 200 U/mL collagenase type I (Worthington Biochemical Corporation, Lakewood, NJ, USA) and 5.4 U/mL protease type XXIV (Sigma-Aldrich, Steinheim, Germany) for 30 min. Solutions were oxygenated with 100 % O_2_ at 37 °C. Supernatant was discarded and the tissue was stirred in protease-free solution 3 times for 5–15 min. Suspensions were centrifuged at 400 rpm for 2 min and cell pellets were resuspended in storage solution containing (in mmol/L) 10 EGTA, 25 glucose, 70 L-glutamic acid potassium salt monohydrate, 10 β-hydroxybutyrat, 20 KCl, 10 KH_2_PO_4_, 40 mannitol, 20 taurine, and 0.1% albumin (pH 7.4). Afterwards, Ca^2+^ was reintroduced by gradually increasing Ca^2+^ concentration to 2 mmol/L and cell suspension was kept at room temperature.

***Action potential (AP) recordings from human and porcine CMs***

APs of human and porcine left and right atrial CMs were measured in current clamp configuration at room temperature. Patch pipettes pulled from borosilicate glass (1B120F-4, World Precision Instruments, Berlin, Germany) with tip resistances ranging from 3 to 8 MΩ were backfilled with internal solution containing (in mmol/L) 134 potassium D-gluconate, 6 NaCl, 1.2 MgCl_2_, 1 MgATP, 10 HEPES (pH 7.2). CMs subjected to AP recordings were placed in external solution containing (in mmol/L) 137 NaCl, 5.4 KCl, 2 CaCl_2_, 1 MgSO_4_, 10 HEPES, 10 glucose (pH 7.3). Following formation of a stable GΩ seal, whole-cell configuration was reached by application of small suction pulses. Pipette capacitance was compensated. CMs were clamped with an average holding current density of -0.95 pA/pF and APs were elicited by injection of 10 brief current pulses (5 ms, 800 pA) at 0.5, 1 or 2 Hz stimulation rate every minute. Dapagliflozin (Biozol, Echingen, Germany) was dissolved in DMSO to a stock solution of 250 mM and stored at -20 °C. After stabilization of the APs, dapagliflozin was added to the chamber at a concentration of 1, 10, or 100 µmol/L. This resulted in a maximal final DMSO concentration of 0.04 % which, in line with previous reports [6], showed no significant effects on AP parameters of atrial CMs (Fig. S4).

***Sodium current recordings from human CMs***

To measure voltage-dependent sodium currents in whole-cell configuration the internal solution contained (in mmol/L) 10 NaCl, 2 CaCl_2_, 3 MgATP, 135 CsCl, 2 TEA-Cl, 5 EGTA, and 0.2 HEPES (pH 7.2) while the external bath solution consisted of (in mmol/L) 20 NaCl, 1.8 CaCl_2_, 1 MgCl_2_, 110 CsCl, 0.001 Nifedipine, 10 Glucose, and 10 HEPES (pH 7.4). CMs were voltage clamped to a holding potential of -100 mV and sodium currents were evoked by application of consecutive 10 mV voltage steps from -100 mV to +20 mV. After stabilization of the peak sodium current, dapagliflozin (1, 10, 100 µmol/L) was added to the chamber.

***Outward potassium current recordings from human CMs***

To separate I_to_ from I_Kur_ by exploiting fast I_to_ recovery from inactivation at 37 °C, double-pulse protocols were used as described [2]. In brief, CMs were voltage clamped to a holding potential of -60 mV. Potassium currents were evoked by two consecutive voltage steps to +50 mV (500 ms), interrupted by a 25 ms repolarizing step to -60 mV. I_to_ was allowed to recover during the brief repolarizing step to -60 mV and was analysed as the area under the curve of the voltage transient during the initial 50 ms of the second clamp step while I_Kur_ was still inactive. Upon stabilization of the current, dapagliflozin (1, 10, 100 µmol/L) was added to the chamber and said measurements were repeated. The internal solution contained (in mmol/L) 60 KCl, 65 K glutamate, 3 K_2_ATP, 0.2 Na_2_GTP, 2 MgCl_2_, 5 EGTA, 5 HEPES (pH 7.2) while the external bath solution consisted of (in mmol/L) 140 NaCl, 5.4 KCl, 1 MgCl_2_, 1 CaCl_2_, 0.33 NaH_2_PO_4_, 5 HEPES, 10 glucose (pH 7.4).

***Multi-electrode array (MEA) electrophysiology***

Electrical activity of human induced pluripotent stem cell (hiPSC)-derived CMs (hiPSC-CMs) was assessed usinag the Maestro Pro MEA system (Axion Biosystems, Atlanta, USA). hiPSC line UMGi014-C clone 14 (isWT1.14) was derived from dermal fibroblasts of a healthy male donor. They were cultured in feeder-free conditions using the integration-free CytoTune iPS 2.0 Sendai Reprogramming Kit (Thermo Fisher Scientific) with reprogramming factors OCT4, KLF4, SOX2, c-MYC. All protocols were approved by the ethics committee of the University Medical Center Göttingen (10/9/15) [3]. CMs were differentiated using established protocols. For MEA analysis, 50.000 hiPSC-CMs per well were seeded in CytoView 24 or 48 well plates around day 90-100 after initiation of differentiation and kept in RPMI 1640 medium (Gibco, Thermo Fisher Scientific) supplemented with B27 (Gibco, Thermo Fisher Scientific). Before the first recording the cells were kept in the MEA system for one hour to obtain a stable environment (37 °C, 5 % CO_2_). The cardiac module was used to continuously record field potentials (FPs) for 10 min at baseline conditions. Afterwards, DMSO or dapagliflozin at 1, 10, 30, or 100 µmol/L was added to the wells and FPs were recorded in the time course after drug application.

***Sodium current recordings from hiPSC-CMs using automated patch clamp (APC)***

Experiments were performed using the automated patch-clamp device, SyncroPatch 384 (Nanion Technologies, Munich, Germany). Negative pressure (150–250 mbar) application attained whole-cell configuration. PatchControl 384 (Nanion Technologies, Munich, Germany) software was used for data acquisition. Thin borosilicate glass 384-well planar chips (1xS-type NPC-384T) were used for all experiments. Recordings were excluded if they showed a seal resistance of <250 MΩ, a peak current of <50 pA, or an R_series_ of >20 MΩ (at 10 mV). Peak sodium currents were measured at 0.5 Hz using a voltage step protocol with a holding potential of -100 mV and a 30 ms test pulse to -20 mV followed by a step to -80 mV for 50 ms at 22–24 °C. Pipette solution contained (in mmol/L): 10 EGTA, 10 HEPES, 10 KCl, 10 NaCl, 110 KF, pH 7.2 (with KOH). Bath solution contained (in mmol/L): 10 HEPES, 140 NaCl, 5 glucose, 4 KCl, 2 CaCl_2_, 1 MgCl_2_, pH 7.4 (with KOH). Offline analysis was performed with DataControl 384 software (Nanion Technologies, Munich, Germany).

***Na_V_1.5 current recordings from transfected Chinese hamster ovary (CHO) cells using APC***

Human Na_V_1.5 cDNA (gene ID: ENSG00000183873) was kindly provided by Steve Goldstein (Brandeis University, Waltham, MA, USA) and subcloned into pMAX, a dual-purpose expression vector containing a CMV promoter for mammalian expression as well as a modified translation initiation sequence (GCC GCC ACC) preceding the first start codon [15]. For generation of the pore mutants SCN5A-F1760A and SCN5A-Y1767A site-directed PCR mutagenesis was performed according to standard methods and the sequences of the resulting plasmids were confirmed by DNA sequencing (Eurofins genomics, Luxembourg). Of note, with reference to the SCN5A sequence variant used in this project, the mutations are located in positions F1759 and Y1766. According to the official nomenclature, however, the common designations F1760 and Y1767 will be used in the following. CHO cells were transfected with pMax^-^SCN5A plasmid DNA using the Lipofectamine 3000 Transfection Kit (Invitrogen, CA, USA) according to the manufacturer´s instructions. APC experiments were performed 24 h after transfection using the SyncroPatch 384 device (Nanion Technologies, Munich, Germany) using the same protocol described above. For IV-recordings the voltage pulse was subsequently increased by 10 mV from -100 to 60 mV. After stabilization of the current at baseline dapagliflozin was washed in at 1 µM and further increased to 10, 100, and 300 µM. To investigate possible direct binding sites of dapagliflozin to Na_V_1.5 two channel mutants were generated (F1760A, Y1767A).

***Molecular Docking***

Molecular docking simulations of dapagliflozin into the recently revealed cryo-EM structure of the rat Na_V_1.5 ortholog (PDB ID: 6UZ0 [7]) were calculated using the SwissDock platform [4] (Swiss Institute of Bioinformatics). Three-dimensional visualizations of the channel structure and docking simulations were generated with PyMOL 2.3 (PyMOL Molecular Graphics System, Schrödinger, LLC, New York, NY, USA).

***RNA expression analysis***

Human atrial tissue samples, collected as described above, were flash frozen in liquid nitrogen and stored at -80 °C. Parts of the tissue samples were homogenized using a TissueRuptor (QIAGEN, Hilden, Germany) system and a total RNA fraction was isolated using TRIzol Reagent (Invitrogen, Thermo Fisher Scientific, Waltham, MA, USA) according to the manufacturer’s instructions. Total RNA of n = 15 SR and n = 15 cAF patients was subjected to poly(A)-enriched bulk RNAseq (Illumina HiSeq, 2x150 bp single index, 20–30 M reads /sample) by a commercial provider (Genewiz Germany GmbH, Leipzig, Germany). The bioinformatic analysis was performed on the Galaxy platform and the DEseq2 algorithm was used for differential expression analysis.

***HCA single cell RNAseq analysis***

Datasets from Litviňuková *et al.* including single-nuclei RNA sequencing data from atrial and ventricular CMs were downloaded from the heart cell atlas webportal (www.heartcellatlas.org) in form of raw count matrices and annotations [12]. A subset consisting of 10,000 atrial and 10,000 ventricular cells was used for downstream analysis with the Seurat package in R-4.2.0, which included normalization and variance stabilization (SCTransform), PCA (RunPCA), UMAP (RunUMAP) and plotting (DimPlot, FeaturePlot, DotPlot).

***Two-electrode voltage clamp measurements***

Two-electrode voltage-clamp measurements were performed on *Xenopus laevis* oocytes 24–72 h after injection of the corresponding cRNA of K_V_1.4, K_V_4.3, K_V_1.5 and Na_V_1.5. For the recordings a bath solution containing (in mmol/L) 101 NaCl, 4 KCl, 2 MgCl_2_, 1.5 CaCl_2_ and 10.0 HEPES, pH 7.4 (with NaOH) was used. The micropipettes were backfilled with a 3 mM KCl-solution (in mmol/L: 102 NaCl, 3 KCl, 2 MgCl_2_, 1.5 CaCl_2_, and 10 HEPES, pH 7.4 (with NaOH)). The micropipettes had a resistance from 1.5 to 3.0 MΩ and were pulled from glass capillaries (GB 100F-10, Science Products, Hofheim, Germany) with a Flaming/Brown P-1000 micropipette puller (Sutter Instruments, Novato, CA, USA). The holding potential was -80 mV, for K_V_1.4, K_V_4.3, and K_V_1.5 currents were elicited with a single step to +40 mv and for Na_V_1.5 with a single step to -30 mV. Leak currents were not subtracted and the experiments were performed at room temperature.

***Data acquisition and statistical analyses***

The Axopatch 200B, Axon Digidata 1550B as well as pCLAMP 10 and 11 (Axon Instruments, Foster City, USA) were used for data acquisition and analysis for manual patch clamp experiments. For automated patch clamp experiments with the SyncroPatch 384 (Nanion Technologies, Munich, Germany) PatchControl 384 (Nanion Technologies, Munich, Germany) was used for data acquisition whereas the DataControl 384 software (Nanion Technologies, Munich, Germany) was used to analyse the data. For MEA electrophysiology data was recorded and analysed using the Maestro Pro MEA system in combination with the Cardiac Module (Axion Biosystems, Atlanta, GA, USA). For the two-electrode voltage clamp measurements, currents were recorded with an OC-725C amplifier (Warner Instruments, Hamden, CT, USA) in conjunction with a Digidata 1322A or Digidata 1550A (Axon Instruments, Foster City, CA, USA) and pCLAMP9 or pCLAMP10 software (Axon Instruments). Further statistical analysis was conducted using Excel (Microsoft, Redmond, WA, USA), R Software (v4.2.0), RStudio 2022.02.2 (Rstudio, Boston, USA) and Prism 9 (GraphPad, La Jolla, CA, USA). Appropriate statistical tests were performed based on the design of the individual experiments as mentioned. *P* < 0.05 was considered as statistically significant.

**Supplementary Figures**


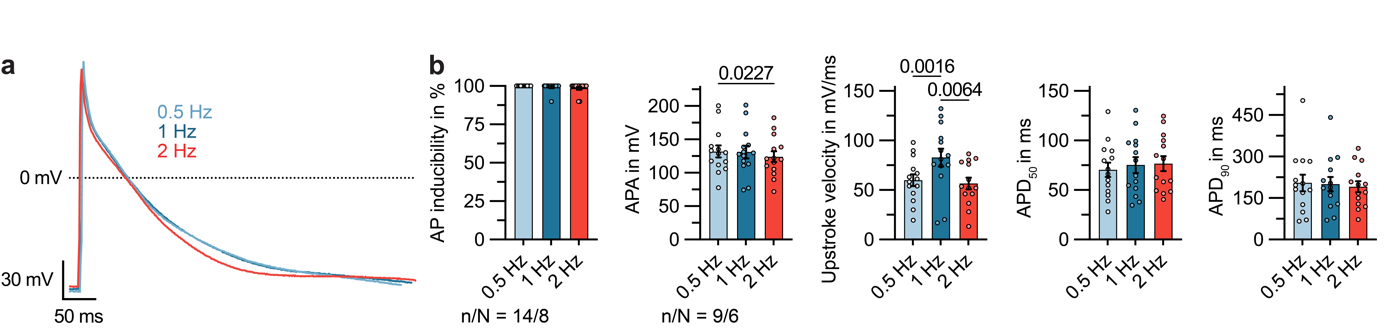


**Fig. S1** Influence of the stimulation frequency on action potential (AP) parameters

**(a)**, Representative APs recorded from porcine atrial cardiomyocytes (CMs) at 0.5 (light blue line), 1 (dark blue line) and 2 Hz (red line) stimulation frequency. The dotted line indicates zero voltage level. **(b)**, AP amplitude (APA), AP duration at 50 % or 90 % repolarization (APD_50_, APD_90_) and resting membrane potential (RMP) of porcine atrial APs at 0.5, 1 and 2 Hz stimulation frequency (n = 18 cells from N = 8 individual animals). Data are shown as mean ± SEM and P-values are derived from repeated measures one-way ANOVA.


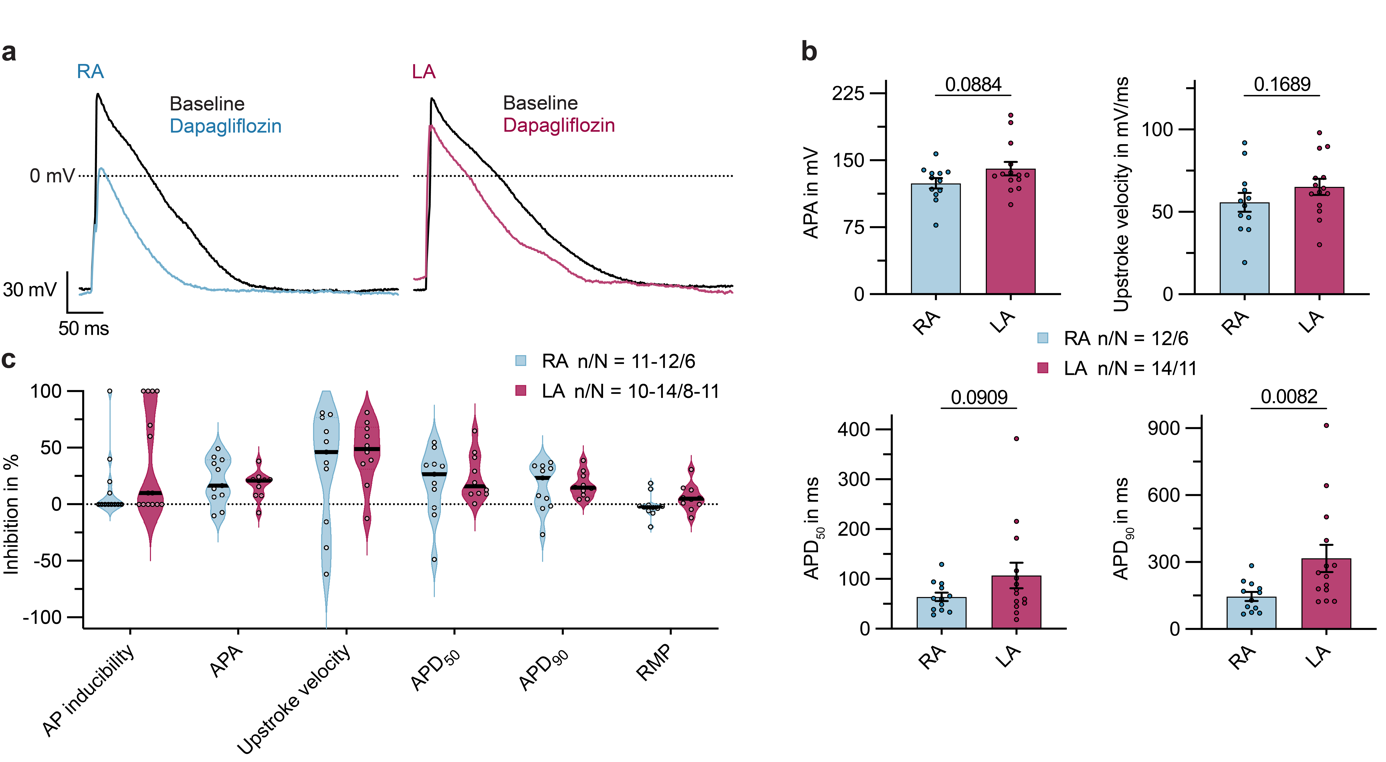


**Fig. S2** Comparison of dapagliflozin effects on left and right atrial porcine action potentials (APs)

**(a)**, Representative AP recordings from porcine cardiomyocytes (CMs) isolated from left atrium (LA) or right atrium (RA) before (baseline) and after application of dapagliflozin (100 µmol/L). Dotted lines indicate zero voltage levels. **(b)**, AP amplitude (APA), upstroke velocity and AP duration at 50 % or 90 % repolarization (APD_50_, APD_90_) of APs recorded from porcine CMs obtained from RA or LA (RA: n/N = 12/6, LA: n/N = 14/11). Data are given as mean ± SEM and P-values are derived from Student´s t-tests. **(c)**, Violin plots showing effects of dapagliflozin on AP inducibility, APA, upstroke velocity, APD_50_, APD_90_ and RMP of RA or LA CMs (RA: n/N = 11–12/6, LA: n/N = 10–14/8–11).


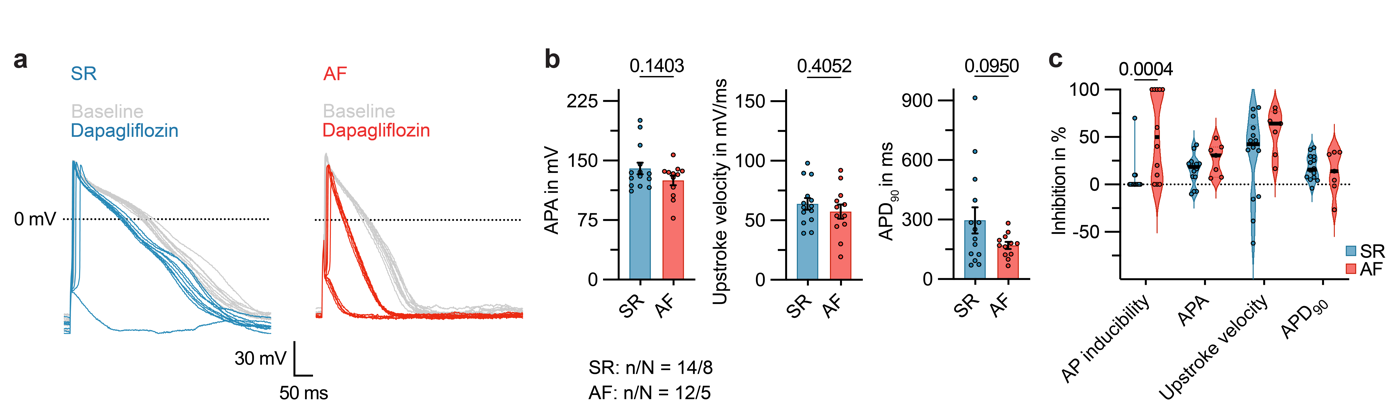


**Fig. S3** Comparison of dapagliflozin effects on cardiomyocytes (CMs) obtained from pigs with sinus rhythm (SR) or atrial fibrillation (AF)

**(a,b,c)** Subanalysis of AP recordings from porcine CMs shown in Fig 1, stratified by rhythm status. **(a)**, Representative APs (elicited by 10 current pulses at a stimulation frequency of 0.5 Hz) recorded from isolated atrial CMs from SR or AF pigs are depicted before and after administration of dapagliflozin (100 µmol/L). **(b)**, AP amplitude (APA), maximum upstroke velocity and AP duration at 90 % repolarization (APD_90_) of atrial CMs derived from pigs with SR or AF (SR: n = 14 cells from N = 8 individual animals; AF: n/N = 12/5). **(c)**, Violin plots comparing dapagliflozin-induced inhibition of AP inducibility (SR: n/N = 14/8; AF: n/N = 12/5) and AP parameters (SR: n/N = 14/8; AF: n/N = 7/3) among atrial CMs isolated from SR or AF pigs.

**
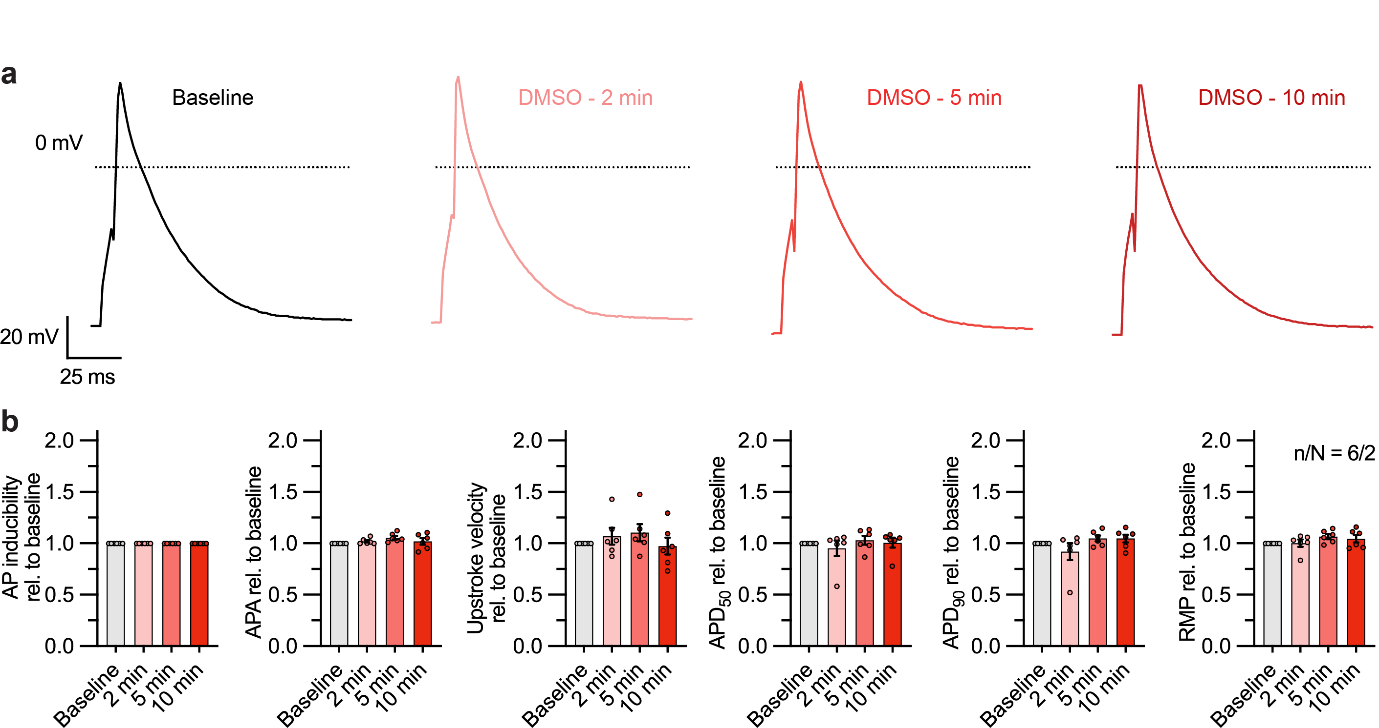
**

**Fig. S4** Solvent control of human atrial action potential (APs) measurements

**(a)**, Representative AP recordings from human cardiomyocytes (CMs) before (baseline) and in the time course after application of dimethylsulfoxide (DMSO) as solvent control at a concentration of 0.04 %, equal to applicated amounts of DMSO during patch clamp measurements with 100 µmol/L dapagliflozin. **(b)**, AP inducibility, AP amplitude (APA), Upstroke velocity, AP duration at 50 % or 90 % repolarization (APD_50_, APD_90_) and resting membrane potential (RMP) of human APs 2, 5 and 10 min after application of DMSO as solvent control (n/N = 6/2) relative to values at baseline. Data are given as mean ± SEM and P-values are derived from RM one-way ANOVA.


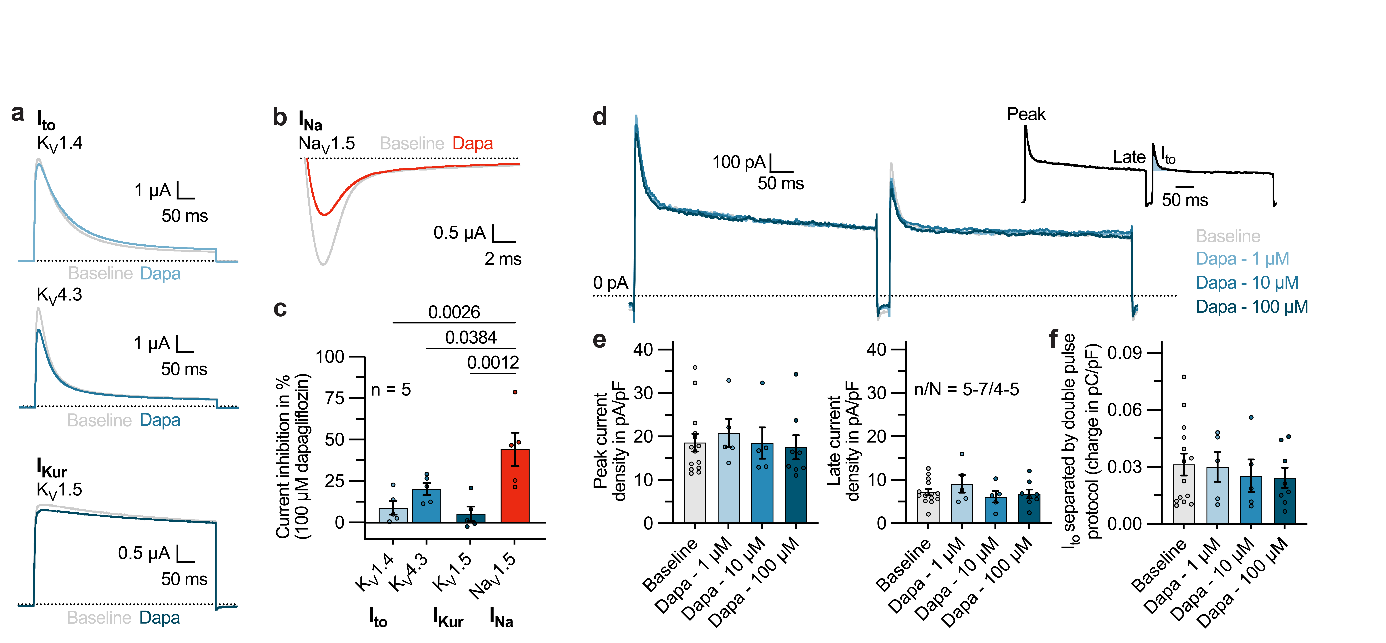


**Fig. S5** Effects of dapagliflozin on outward potassium currents

**(a-c)**, Two-electrode voltage clamp recordings of human potassium and sodium channels expressed in *Xenopus laevis* oocytes. **(a)**, Representative human K_V_1.4, K_V_4.3 (I_to_) and K_V_1.5 (I_Kur_) current traces before (baseline) and after administration of dapagliflozin (100 µmol/L). **(b)**, Representative human Na_V_1.5 current traces before (baseline) and after administration of dapagliflozin (100 µmol/L). **(c)**, Effect of dapagliflozin (100 µmol/L) on the respective potassium channels, compared to the inhibitory effect on Na_V_1.5 (n = 5). **(d–f),** Patch clamp recordings of outward potassium currents on human atrial cardiomyocytes (CMs). **(d)**, Representative current traces recorded from CMs under baseline conditions and after administration of increasing dapagliflozin concentrations (1, 10, 100 µmol/L). **(e)**, Peak current and late current densities at baseline and after administration of dapagliflozin (1, 10, 100 µmol/L, n/N = 5–7/4–5). **(f)**, I_to_ charge, separated by a double pulse protocol by exploiting the fast recovery from inactivation of I_to_ as opposed to I_Kur_, at baseline and after administration of dapagliflozin (1, 10, 100 µmol/L, n/N = 5–-7/4–5). Data are given as mean ± SEM and P-values are derived from RM one-way ANOVA.


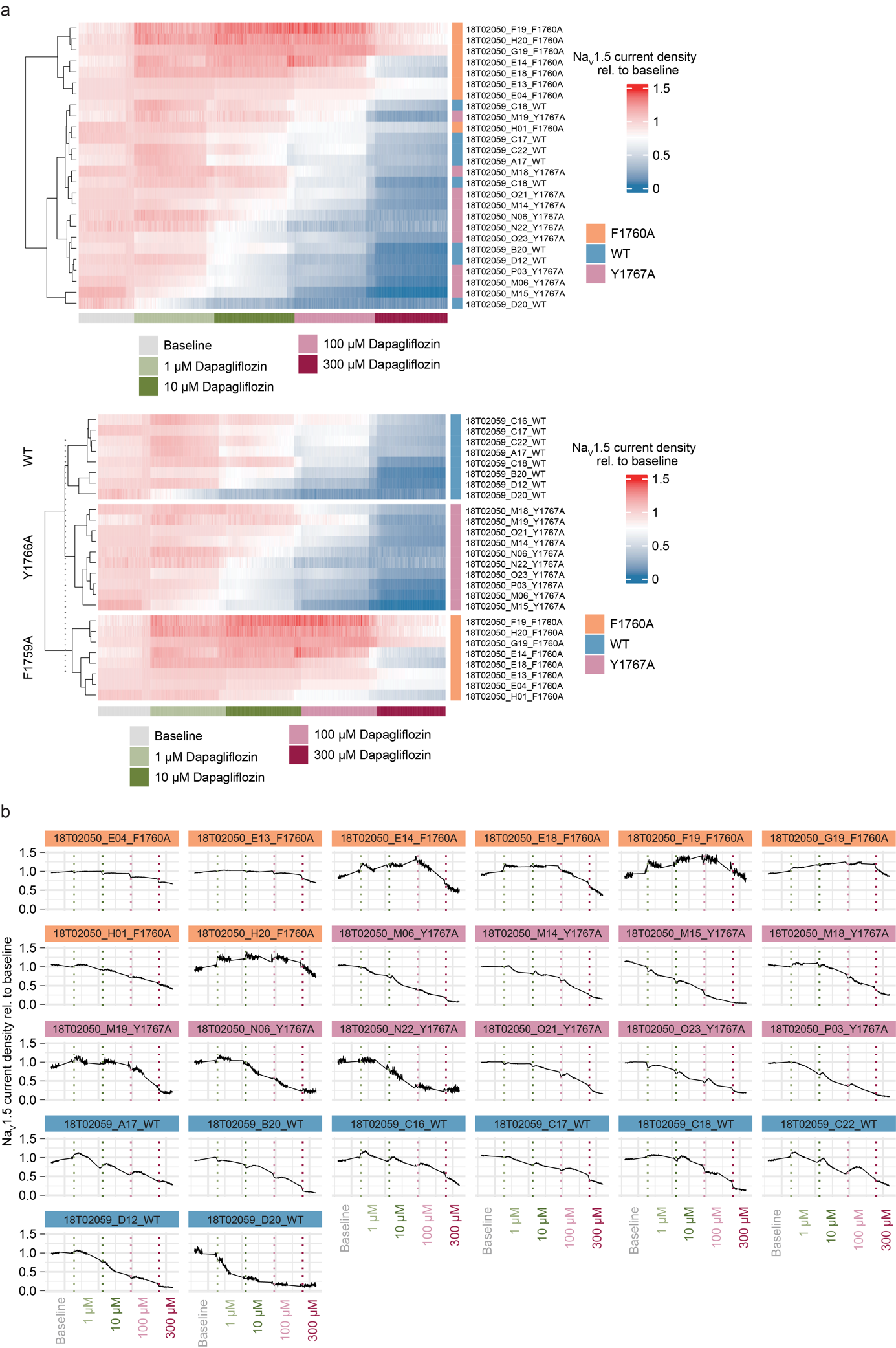


**Fig. S6** Cluster analysis of dapagliflozin effects on Na_V_1.5 currents in Chinese hamster ovary (CHO) cells transfected with SCN5A wild-type (WT), Y1767A or F1760A

**(a)**, Heatmaps display changes in the Na_V_1.5 current densities relative to baseline values recorded from CHO cells transiently transfected with SCN5A WT, Y1767A or F1760A during stepwise increase of dapagliflozin concentrations (1–300 µmol/L). Two main clusters of cells regarding the dapagliflozin effect on Na_V_1.5 current densities are indicated (WT: n = 8, Y1767A: n = 10, F1760A: n = 10). **(b)**, Time course of Na_V_1.5 peak current densities measured at -20 mV from CHO cells transfected with SCN5A WT, Y1767A or F1760A during gradual increase of the dapagliflozin concentration (1–300 µmol/L). Na_V_1.5 current densities are shown relative to current densities at baseline (WT: n = 8, Y1767A: n = 10, F1760A: n = 10).


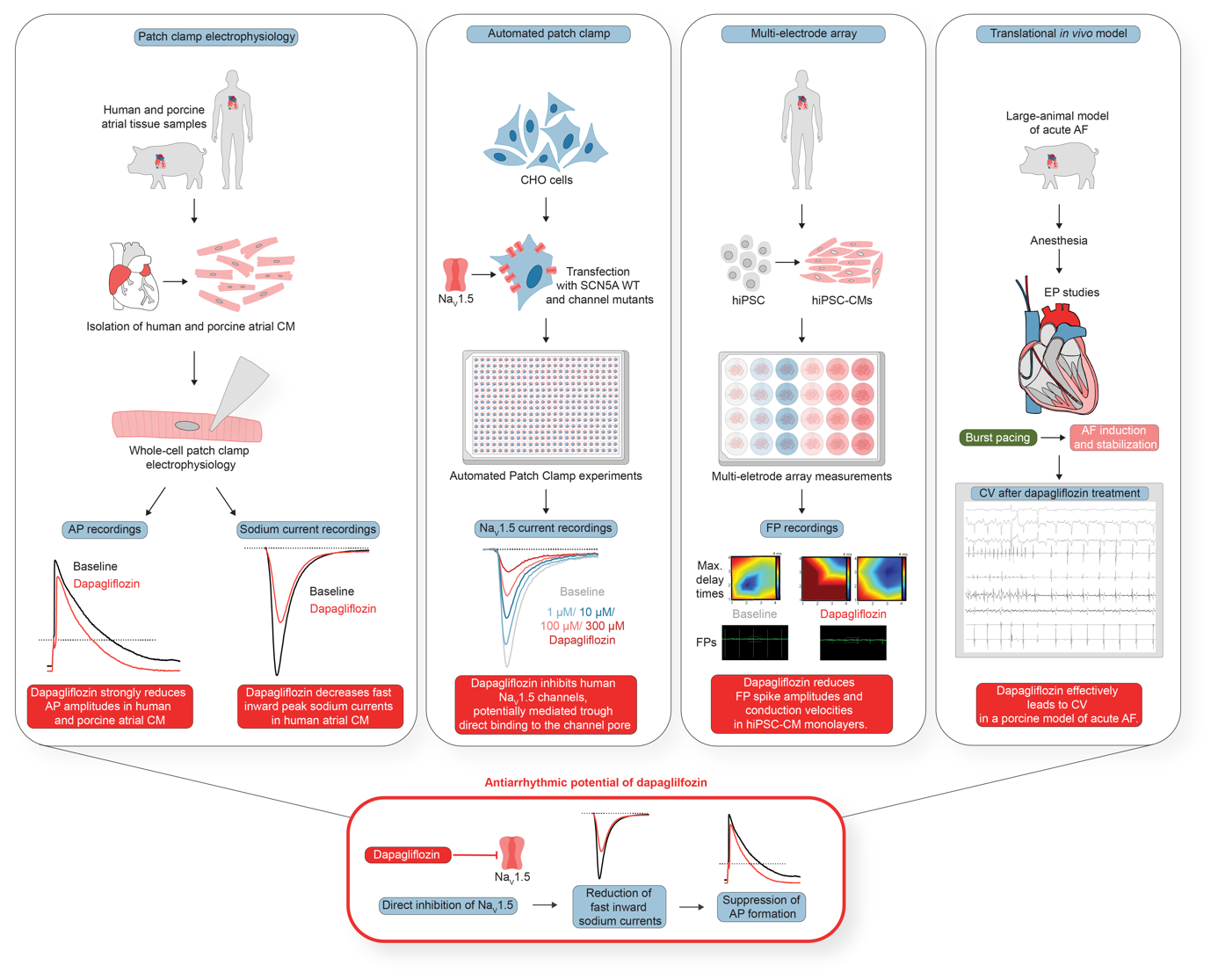


**Fig. S7** Summarizing Figure: The antiarrhythmic potential of dapagliflozin

**Supplementary Tables**

| **Animal model** | **SGLT inhibitor** | **Dose** | **Time**  **period of treatment** | **Electrophysiological endpoint (if any)** | **Citation** |
| --- | --- | --- | --- | --- | --- |
| Mitral regurgitation  model in rats | Dapagliflozin | 10 mg/kg  per day  (orally) | 6 weeks | Lower inducibility  and shorter duration  of pacing-induced  AF episodes | [11] |
| High fat diet in  Spontaneous  hypertensive rats | Dapagliflozin | 20 mg/kg  per day  (orally) | 12 weeks | - | [9] |
| Neurogenic  hypertensive mouse  model | Dapagliflozin | 40 mg/kg  every 2 days  (orally) | 2 weeks | - | [5] |
| Diabetic  cardiomyopathy in  mice (db/db  mice on high-fat  western diet) | Empagliflozin | 150 mg/kg  per day  (orally) | 4.5 weeks | - | [13] |
| Inflammatory  /septic  cardiomyopathy in  mice (LPS  induced) | Empagliflozin | 5 mg/kg  (i.p.) | Single time point | - | [8] |
| Heart failure with  preserved ejection  fraction in rats  (ZSF-1 obese rats) | Sotagliflozin | 30 mg/kg  per day  (orally) | 6 weeks | Reduced magnitude  of arrhythmic  spontaneous Ca^2+^  release events in  isolated left atrial  cardiomyocytes | [1] |
| Diabetic rats  (streptozotocin-  induced) | Empagliflozin | 10 mg/kg  (orally) | 4 weeks | - | [10] |

**Table S1** Translational animal models studying the cardiac effects of SGLT inhibitors

Translation animal models studying the cardiac effects of SGLT inhibitors are listed together with the dose, route of administration and treatment time.

**Supplementary References**

1. Bode D, Semmler L, Wakula P, Hegemann N, Primessnig U, Beindorff N, Powell D, Dahmen R, Ruetten H, Oeing C, Alogna A, Messroghli D, Pieske BM, Heinzel FR, Hohendanner F (2021) Dual SGLT-1 and SGLT-2 inhibition improves left atrial dysfunction in HFpEF. Cardiovasc Diabetol 20:7 doi:10.1186/s12933-020-01208-z

2. Christ T, Wettwer E, Voigt N, Hála O, Radicke S, Matschke K, Várro A, Dobrev D, Ravens U (2008) Pathology-specific effects of the IKur/Ito/IK,ACh blocker AVE0118 on ion channels in human chronic atrial fibrillation. Br J Pharmacol 154:1619-1630 doi:10.1038/bjp.2008.209

3. Cyganek L, Tiburcy M, Sekeres K, Gerstenberg K, Bohnenberger H, Lenz C, Henze S, Stauske M, Salinas G, Zimmermann WH, Hasenfuss G, Guan K (2018) Deep phenotyping of human induced pluripotent stem cell-derived atrial and ventricular cardiomyocytes. JCI Insight 3 doi:10.1172/jci.insight.99941

4. Grosdidier A, Zoete V, Michielin O (2011) SwissDock, a protein-small molecule docking web service based on EADock DSS. Nucleic Acids Res 39:W270-277 doi:10.1093/nar/gkr366

5. Herat LY, Magno AL, Rudnicka C, Hricova J, Carnagarin R, Ward NC, Arcambal A, Kiuchi MG, Head GA, Schlaich MP, Matthews VB (2020) SGLT2 Inhibitor-Induced Sympathoinhibition: A Novel Mechanism for Cardiorenal Protection. JACC Basic Transl Sci 5:169-179 doi:10.1016/j.jacbts.2019.11.007

6. Hyun SW, Kim BR, Hyun SA, Seo JW (2017) The assessment of electrophysiological activity in human-induced pluripotent stem cell-derived cardiomyocytes exposed to dimethyl sulfoxide and ethanol by manual patch clamp and multi-electrode array system. J Pharmacol Toxicol Methods 87:93-98 doi:10.1016/j.vascn.2017.03.003

7. Jiang D, Zhang J, Xia Z (2022) Structural Advances in Voltage-Gated Sodium Channels. Front Pharmacol 13:908867 doi:10.3389/fphar.2022.908867

8. Koyani CN, Plastira I, Sourij H, Hallström S, Schmidt A, Rainer PP, Bugger H, Frank S, Malle E, von Lewinski D (2020) Empagliflozin protects heart from inflammation and energy depletion via AMPK activation. Pharmacol Res 158:104870 doi:10.1016/j.phrs.2020.104870

9. Lee HC, Shiou YL, Jhuo SJ, Chang CY, Liu PL, Jhuang WJ, Dai ZK, Chen WY, Chen YF, Lee AS (2019) The sodium-glucose co-transporter 2 inhibitor empagliflozin attenuates cardiac fibrosis and improves ventricular hemodynamics in hypertensive heart failure rats. Cardiovasc Diabetol 18:45 doi:10.1186/s12933-019-0849-6

10. Lee TI, Chen YC, Lin YK, Chung CC, Lu YY, Kao YH, Chen YJ (2019) Empagliflozin Attenuates Myocardial Sodium and Calcium Dysregulation and Reverses Cardiac Remodeling in Streptozotocin-Induced Diabetic Rats. Int J Mol Sci 20 doi:10.3390/ijms20071680

11. Lin YW, Chen CY, Shih JY, Cheng BC, Chang CP, Lin MT, Ho CH, Chen ZC, Fisch S, Chang WT (2021) Dapagliflozin Improves Cardiac Hemodynamics and Mitigates Arrhythmogenesis in Mitral Regurgitation-Induced Myocardial Dysfunction. J Am Heart Assoc 10:e019274 doi:10.1161/jaha.120.019274

12. Litviňuková M, Talavera-López C, Maatz H, Reichart D, Worth CL, Lindberg EL, Kanda M, Polanski K, Heinig M, Lee M, Nadelmann ER, Roberts K, Tuck L, Fasouli ES, DeLaughter DM, McDonough B, Wakimoto H, Gorham JM, Samari S, Mahbubani KT, Saeb-Parsy K, Patone G, Boyle JJ, Zhang H, Zhang H, Viveiros A, Oudit GY, Bayraktar OA, Seidman JG, Seidman CE, Noseda M, Hubner N, Teichmann SA (2020) Cells of the adult human heart. Nature 588:466-472 doi:10.1038/s41586-020-2797-4

13. Moellmann J, Klinkhammer BM, Droste P, Kappel B, Haj-Yehia E, Maxeiner S, Artati A, Adamski J, Boor P, Schütt K, Lopaschuk GD, Verma S, Marx N, Lehrke M (2020) Empagliflozin improves left ventricular diastolic function of db/db mice. Biochim Biophys Acta Mol Basis Dis 1866:165807 doi:10.1016/j.bbadis.2020.165807

14. Schmidt C, Wiedmann F, Beyersdorf C, Zhao Z, El-Battrawy I, Lan H, Szabo G, Li X, Lang S, Korkmaz-Icöz S, Rapti K, Jungmann A, Ratte A, Müller OJ, Karck M, Seemann G, Akin I, Borggrefe M, Zhou XB, Katus HA, Thomas D (2019) Genetic Ablation of TASK-1 (Tandem of P Domains in a Weak Inward Rectifying K(+) Channel-Related Acid-Sensitive K(+) Channel-1) (K(2P)3.1) K(+) Channels Suppresses Atrial Fibrillation and Prevents Electrical Remodeling. Circ Arrhythm Electrophysiol 12:e007465 doi:10.1161/circep.119.007465

15. Schmidt C, Wiedmann F, El-Battrawy I, Fritz M, Ratte A, Beller CJ, Lang S, Rudic B, Schimpf R, Akin I, Karck M, Borggrefe M, Katus HA, Zhou XB, Thomas D (2018) Reduced Na(+) Current in Native Cardiomyocytes of a Brugada Syndrome Patient Associated With β-2-Syntrophin Mutation. Circ Genom Precis Med 11:e002263 doi:10.1161/circgen.118.002263

16. Wiedmann F, Beyersdorf C, Zhou XB, Kraft M, Foerster KI, El-Battrawy I, Lang S, Borggrefe M, Haefeli WE, Frey N, Schmidt C (2020) The Experimental TASK-1 Potassium Channel Inhibitor A293 Can Be Employed for Rhythm Control of Persistent Atrial Fibrillation in a Translational Large Animal Model. Front Physiol 11:629421 doi:10.3389/fphys.2020.629421

17. Wiedmann F, Beyersdorf C, Zhou XB, Kraft M, Paasche A, Jávorszky N, Rinné S, Sutanto H, Büscher A, Foerster KI, Blank A, El-Battrawy I, Li X, Lang S, Tochtermann U, Kremer J, Arif R, Karck M, Decher N, van Loon G, Akin I, Borggrefe M, Kallenberger S, Heijman J, Haefeli WE, Katus HA, Schmidt C (2022) Treatment of atrial fibrillation with doxapram: TASK-1 potassium channel inhibition as a novel pharmacological strategy. Cardiovasc Res 118:1728-1741 doi:10.1093/cvr/cvab177
